# Audio–visual and olfactory–visual integration in healthy participants and subjects with autism spectrum disorder

**DOI:** 10.1002/hbm.24715

**Published:** 2019-07-13

**Authors:** Susanne Stickel, Pauline Weismann, Thilo Kellermann, Christina Regenbogen, Ute Habel, Jessica Freiherr, Natalya Chechko

**Affiliations:** ^1^ Department of Psychiatry, Psychotherapy and Psychosomatics Faculty of Medicine, RWTH Aachen Aachen Germany; ^2^ Institute of Neuroscience and Medicine: JARA‐Institute Brain Structure Function Relationship (INM 10) Research Center Jülich Jülich Germany; ^3^ Department of Psychiatry and Psychotherapy Friedrich‐Alexander‐Universität Erlangen‐Nürnberg Erlangen Germany; ^4^ Department of Clinical Neuroscience Karolinska Institutet Stockholm Sweden; ^5^ Sensory Analytics Fraunhofer Institute for Process Engineering and Packaging IVV Freising Germany

**Keywords:** auditory–visual integration, autism spectrum disorder, dynamic causal modeling, fMRI, multisensory integration, olfactory–visual integration

## Abstract

The human capacity to integrate sensory signals has been investigated with respect to different sensory modalities. A common denominator of the neural network underlying the integration of sensory clues has yet to be identified. Additionally, brain imaging data from patients with autism spectrum disorder (ASD) do not cover disparities in neuronal sensory processing. In this fMRI study, we compared the underlying neural networks of both olfactory–visual and auditory–visual integration in patients with ASD and a group of matched healthy participants. The aim was to disentangle sensory‐specific networks so as to derive a potential (amodal) common source of multisensory integration (MSI) and to investigate differences in brain networks with sensory processing in individuals with ASD. In both groups, similar neural networks were found to be involved in the olfactory–visual and auditory–visual integration processes, including the primary visual cortex, the inferior parietal sulcus (IPS), and the medial and inferior frontal cortices. Amygdala activation was observed specifically during olfactory–visual integration, with superior temporal activation having been seen during auditory–visual integration. A dynamic causal modeling analysis revealed a nonlinear top‐down IPS modulation of the connection between the respective primary sensory regions in both experimental conditions and in both groups. Thus, we demonstrate that MSI has shared neural sources across olfactory–visual and audio–visual stimulation in patients and controls. The enhanced recruitment of the IPS to modulate changes between areas is relevant to sensory perception. Our results also indicate that, with respect to MSI processing, adults with ASD do not significantly differ from their healthy counterparts.

## 1 INTRODUCTION

The mechanism behind the interpretation and encoding of our immediate environment usually depends on multisensory integration (MSI) processes, in which different sensory channels processing information independent of one another are integrated into a unique and coherent picture. Thus, it is by combining information from complex, cross‐modal stimuli, that the human brain produces unitary perception (Stein & Stanford, 2008). The inferior parietal sulcus (IPS) and the superior temporal sulcus (STS) have been found to play key roles in mediating information exchange between the primary sensory cortices to facilitate conclusions regarding the identity of particular objects (Beauchamp, Lee, Argall, & Martin, 2004; Fairhall & Macaluso, 2009; Grefkes, Weiss, Zilles, & Fink, 2002; Regenbogen et al., 2017; Sereno & Huang, 2014).

MSI is most effective when physiologically weak cues are presented. In other words, unisensory, highly salient stimuli are easily detected and identified while physiologically weak sensory signals do not trigger a response and need to be amplified by another stimulus (Holmes, 2009; Stein & Stanford, 2008). According to this so‐called principle of inverse effectiveness, the combination of weak stimuli, which are not effective on their own, far exceeds the sum of their individual responses, thus producing the greatest enhancement in MSI through a superadditive combination (Nagy, Eördegh, Paróczy, Márkus, & Benedek, 2006; Stein, Stanford, & Rowland, 2014). In audio–visual integration studies, for instance, the presentation of degraded auditory stimuli has been found to enable MSI (Regenbogen et al., 2017). In humans, nontrigeminal odors produce a relatively weak subjective experience compared to other senses such as hearing or seeing (Licon, Manesse, Dantec, Fournel, & Bensafi, 2018; Moessnang et al., 2013). However, in this context, it must be borne in mind that, to date, there have been only a handful of studies pertaining to olfactory–visual integration (e.g., Gottfried & Dolan, 2003; Lundström, Regenbogen, Ohla, & Seubert, 2018; Ripp et al., 2018; Sijben, Hoffmann‐Hensel, Rodrigues‐Raecke, Haarmeier, & Freiherr, 2018), with multisensory fMRI research in humans focusing predominantly on the integration of auditory and/or somatosensory cues and visual information (e.g., Chen et al., 2015; Regenbogen et al., 2017; Renier et al., 2009). Moreover, none of the previous studies sought to investigate olfactory–visual MSI in comparison to audio–visual MSI; thus, it is yet to be determined which brain networks are generally involved in multisensory processing (amodal) and which are specific to the type of multimodal sensory stimulation (sensory specific).

Compared to other senses such as hearing or seeing, smelling has only recently been considered important due to its complex effects on behavior and mood (Moscavitch, Szyper‐Kravitz, & Shoenfeld, 2009). A close link between olfactory processing and emotional processing can be explained by the fact that both processes share common anatomical structures in the so‐called limbic system (Habel et al., 2007; Rolls, Grabenhorst, & Parris, 2010; Royet, Plailly, Delon‐Martin, Kareken, & Segebarth, 2003; Seubert, Freiherr, Djordjevic, & Lundström, 2013; Zald & Pardo, 1997). Apart from its relevance to MSI processes, olfactory dysfunctions can also help shed light on the underlying functional and structural integrity of the brain regions involved in neuropsychiatric diseases (Martzke, Kopala, & Good, 1997), given that there is evidence of impairments in olfactory perception in conditions such as schizophrenia, autism spectrum disorder (ASD), Parkinson's disease, and Alzheimer's disease (e.g., Attems, Walker, & Jellinger, 2014; Rozenkrantz et al., 2015; Turetsky, Hahn, Borgmann‐Winter, & Moberg, 2009).

ASD is a neurodevelopmental disorder with limited functions in social interaction along with stereotyped activities and repetitive behavior (American Psychiatric Association, 2013). Past research on ASD has revolved around deficits in social interaction, language, communication, and recognition of affect. Lately, the focus has shifted toward more basic sensory abnormalities associated with autism, which have been incorporated as additional diagnostic criteria in the last updated version of the Diagnostic and Statistical Manual of Mental Disorders (DSM‐5; American Psychiatric Association, 2013). In this context, individuals with ASD can show hypersensitivity or hyposensitivity to sensory input or unusual interest in sensory features of the environment, such as “apparent indifference to pain/temperature, adverse response to specific sounds or textures, excessive smelling or touching of objects, visual fascination with lights or movement” (American Psychiatric Association, 2013).

Abnormalities in ASD are reported in all sensory domains (Thye, Bednarz, Herringshaw, Sartin, & Kana, 2018) and are linked to a disturbed underlying neural processing (Kern et al., 2007). For example, compared to typically developing children, children with ASD have a delay in the identification of speech syllables in lip‐reading tasks and are less likely to integrate the audio–visual information obtained by the McGurk effect (Taylor, Isaac, & Milne, 2010). Another study has demonstrated that these integration deficits are amplified with increasing background noise (Foxe et al., 2015). The underlying reasons seem to be an interregional communication disturbances (Beker, Foxe, & Molholm, 2018). Children (7–16 years) with autism, for instance, show a weaker multisensory facilitation of behavior toward audio–visual stimuli and ineffective neural integration (Brandwein et al., 2013). All of these observations on behavioral and neural levels support the theory of weak central coherence in ASD, which suggests that ASD renders individuals incapable of embedding disparate pieces of information into a comprehensive whole (Frith & Happé, 1994). At the same time, however, patients' aptitude to process the details of stimuli may be enhanced compared to their healthy counterparts (Foxton et al., 2003). Thus, there is evidence in ASD (especially in children with ASD) of impaired processing of complex information as opposed to unaffected processing of simple information (Bertone, Mottron, Jelenic, & Faubert, 2003; Bertone, Mottron, Jelenic, & Faubert, 2005). This conclusion, however, is challenged by a recent meta‐analysis, which suggests, summarizing studies on a variety of audio–visual MSI tasks in ASD, that differences between patients and typically developing individuals increasingly balance out with age (Feldman et al., 2018).

Despite increased attention to research on olfactory processing in ASD, studies in the subject are still scarce. The initial findings show that children with autism do not adjust their sniff magnitude regardless of the odor valence as typically developing children do (Rozenkrantz et al., 2015). However, the findings with respect to olfactory processing in ASD are partly contradictory. While compared to controls, adults with ASD have an impaired ability to identify odors (Bennetto, Kuschner, & Hyman, 2007; Suzuki, 2003; Tonacci et al., 2015; Wicker, Monfardini, & Royet, 2016), a greater olfactory sensitivity has been found to correlate with a higher number of autistic traits (Ashwin et al., 2014), supporting the notion of enhanced perceptual functioning in ASD and similar accounts (see Larsson, Tirado, & Wiens, 2017 for a meta‐analysis; Mottron, Dawson, Soulières, Hubert, & Burack, 2006).

It thus remains unclear whether difficulties in MSI can be observed in ASD adults, especially in those with Asperger's. In addition, while behavior‐oriented audio–visual integration processes in ASD have been investigated, there is yet to be any in‐depth study of the integration processes based on olfactory and visual system stimulation in ASD. To the best of our knowledge, there is no fMRI study of either mechanism in ASD. The condition is associated with an impaired ability to integrate multisensory information, but it remains unknown whether peripheral or central nervous processing of multimodal stimuli is affected.

The aim of our study, therefore, was to compare the neuronal processes during audio–visual and olfactory–visual integration. Neural activation patterns were examined using two independent experiments, one with an audio–visual stimulus combination and the other with an olfactory–visual stimulus combination. On the basis of the available literature, we assumed that both integration paradigms would activate a common network linked to MSI, involving the IPS as a central cross‐modal “hub” of MSI (Grefkes et al., 2002; Regenbogen et al., 2017; Sereno & Huang, 2014). Based on previous findings (e.g., Regenbogen et al., 2017), we hypothesized the involvement of the IPS in a top‐down modulation in both cross‐modal settings studied by means of dynamic causal modeling (DCM; Stephan et al., 2010). In addition to an underlying common network, we expected sensory‐specific differences with respect to the processing of different sensory modalities. More specifically, we sought to explore whether high‐order MSI is impaired in adult subjects with ASD as had been observed previously at the behavioral level in children with ASD (Stevenson et al., 2014), and if the impairment depends on the sensory nature of multimodal processing. Thus, we hypothesized an overlap of the networks involved in sensory‐specific multimodal integration in adults with ASD and healthy controls (HCs), postulating also that adults with ASD would exhibit a lower degree of integration likely on account of reduced neural processing of amodal stimulation.

## 2 METHODS

### 2.1 Participants

Written informed consent was obtained from all participants and the study, approved by the local ethics committee, conformed to the ethical standards of the Helsinki declaration.

Participants in the ASD group were outpatients with Asperger's syndrome diagnosed at the Medical Faculty RWTH Aachen and characterized by information from the German version of the Autism Diagnostic Observation Schedule (ADOS) (Rühl, Bölte, Feineis‐Matthews, & Poustka, 2004). Age‐ and gender‐matched control subjects (HC) were healthy adults without psychiatric disorders, identified by the short version of the German Structured Clinical Interview for DSM 4 Disorders (Wittchen, Zaudig, & Frydrich, 1997). To control for normosmia, the MONEX‐40 olfactory identification test was performed with each participant having to identify 40 common odors among four descriptors each (Freiherr et al., 2012).

To avoid confounding psychological effects on olfactory perception and sensitivity (Atanasova et al., 2008; Burón, Bulbena, & Bulbena‐Cabré, 2015; Croy et al., 2014; Naudin & Atanasova, 2014; Yuan & Slotnick, 2014) and to assess clinical impairment in healthy participants, the following questionnaires were used: the German version of the Beck Depression Inventory II (BDI) (Hautzinger, Keller, & Kühner, 2006), autism questionnaire (Baron‐Cohen, Wheelwright, Skinner, Martin, & Clubley, 2001), and a German questionnaire for personality style and disorders (Persönlichkeits‐Stil und Störungs‐Inventar; Kuhl & Kazén, 2009). Additional tests were conducted in order to compare the two groups: verbal intelligence quotient (IQ) using the German Wortschatztest (Schmidt & Metzler, 1992), executive functions via the Trail Making Test (Reitan, 1992), word fluency using the German Regensburger Wortflüssigkeitstest (Aschenbrenner, Tucha, & Lange, 2000), Emotion Recognition 40 (Gur et al., 2002; Hoheisel & Kryspin‐Exner, 2005), and the German version of the Wechsler Memory Scale (Härtling et al., 2000). None of the participants reported nasal congestion or diseases of the respiratory system.

In total, 24 patients with ASD and 20 with HC were examined. Data from four patients with ASD and two HC had to be discarded due to movement artifacts in the MRI. Additionally, two patients did not complete the functional magnetic resonance imaging (fMRI) measurement. Imaging data of 18 patients with ASD (seven females, mean age in years: 30.72 [*SD* = 8.89]; ADOS range: 5–13 [median = 9]) and 17 healthy participants (six female, mean age in years: 28.84 [*SD* = 9.88]) were used for further analyses. The groups did not differ significantly with respect to age (*t*(33)= −0.837, *p* = .409). Although the ASD group has a significantly higher depressive symptomology (see Table 1) and deficits in olfactory functions can be seen as a marker for depression (Croy et al., 2014), we found no significant correlation between the olfactory identification test and BDI symptoms (*r* = −.225, *p* = .201). Additionally, the groups did not differ with regard to normosmia either when depressive symptoms were controlled for (*F*(1,31)= 0.319, *p* = .576, η^2^ = .01) or when they were not (*t*(33) = 1.21, *p* = .253). Therefore, in the ASD group, depressive symptoms can be deemed to have no effect on olfactory performance.

**Table 1:** Psychometric results of patients with ASD and HCs

	ASD mean (*SD*)	HC mean (*SD*)	*t* Test	*p*
Beck's depression inventory (total)	14.71 (8.72)	4.42 (4.79)	−4.45	.001
AQ (raw value)	36.23 (10.65)	11.21 (5.37)	−9.05	.001
Emotion recognition (total identified)	28.41 (4.32)	32.88 (1.96)	3.89	.001
Verbal IQ	103.50 (12.29)	111.76 (9.53)	2.17	.038
Word fluency (raw value)	55.46 (11.31)	55.36 (9.02)	−0.28	.978
Executive functions (total seconds)	76.17 (20.61)	89.56 (1.82)	2.67	.012
Wechsler memory scale (raw value)	24.88 (6.33)	29.67 (9.28)	1.60	.120

Abbreviations: AQ, autism questionnaire; ASD, autism spectrum disorder; HC, healthy control.

During the (semi‐)structured ADOS interview, participants with ASD are asked about hypersensitivity or hyposensitivity to sensory input and are routinely observed with respect to unusual sensory interests or habits. A total of seven participants reported sensory differences. Four of them reported that they had a strong aversion to the sensation of touch, including that of clothing on the skin. One participant reported to be sensitive to light and noise, while three were observed to repeatedly or constantly touch and stroke smooth objects (i.e., tables). None of the participants reported anomalies with respect to olfactory impressions.

Details of the psychological assessments and performance tests in both groups are listed in Table 1.

### 2.2 Olfactory stimulation

Olfactory stimuli were delivered birhinally using a computer‐controlled, air dilution olfactometer (Lundström, Gordon, Alden, & Boesveldt, 2011). The air supply was conducted by means of an external, high‐pressure air cylinder. Two pleasant odors, “rose” (2‐phenylethanol, Burghart Messtechnik, Wedel, Germany) and “peach,” (peach D40 Reco AB8409; Givaudan UK Ltd., Ashford, UK), and two unpleasant odors, “manure” (3‐methylindole 98% M51458; Sigma‐Aldrich) and “dirty socks” (isovaleric acid 129542; Sigma‐Aldrich Chemie GmbH, Munich, Germany), were presented. As baseline stimulus, the odorless propylene glycol (Hersteller) was turned on whenever no odor was presented. The odors were delivered for 2,200 ms with a continuous airflow of 3.0 L/min through 8‐m long individual Teflon tubes, which were integrated in a special distributor built in the scientific workshop (Medical Faculty RTWH Aachen University). The distributer, terminating in two reusable, autoclavable Teflon nosepieces, was fixed with a nonadhesive, hypoallergenic tape above the upper lip. For olfactory stimulation, the nosepieces were inserted into the subject's nose.

**Figure 1:**
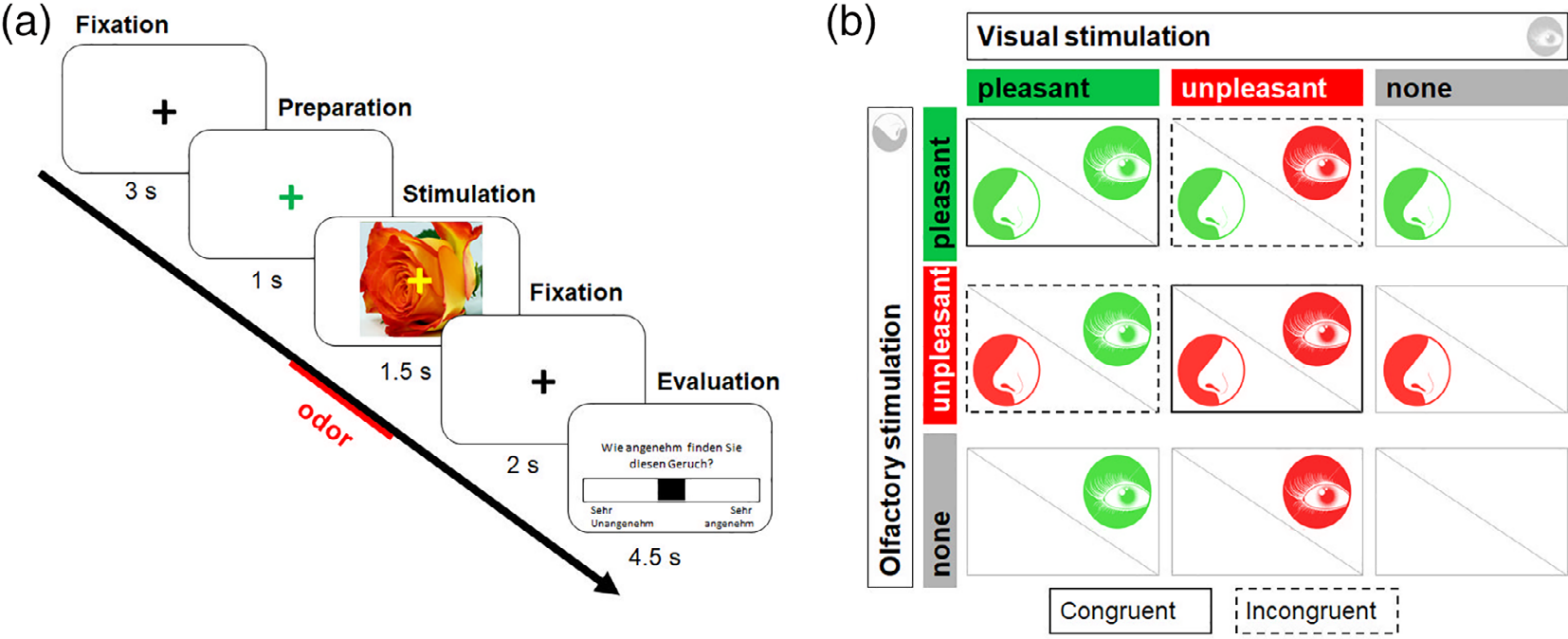
(a) Experimental procedure of the functional tasks. A black fixation cross turned green to indicate upcoming stimuli. Unimodal or bimodal combinations were presented for 1,500 ms. Participants had to rate the pleasantness of the odor or the object, or the sound and the object, respectively. (b) Experimental conditions for olfactory–visual stimulation in a 3 × 3 factorial design. Conditions for auditory–visual stimulation were combined in the same manner [Color figure can be viewed at http://wileyonlinelibrary.com]

The visual stimuli consisted of two pleasant und two unpleasant colored photograph categories of odor‐matching objects. To prevent sensory habituation and to increase object saliency, two images of each visual category were used (Figure 1a).

### 2.3 Auditory stimulation

The auditory stimuli were presented via MRI‐compatible headphones. Pleasant and unpleasant sounds were recorded and tested in an independent pilot group to evaluate the pleasantness of the sounds. Two pleasant sounds (working coffee machine and fireplace) and two unpleasant sounds (toilet flush and vomiting sound) were combined with relevant images. Vomiting was coupled with rotten fruits, which had the highest ranking in the pilot group with respect to cohesiveness.

### 2.4 Experimental paradigms

In the olfactory–visual paradigm, two pleasant (rose, peach) and two unpleasant (manure, dirty socks) stimuli were presented in unimodal fashion (only odor, only visual) or were paralleled with either congruent or incongruent images or odors. A baseline condition was implemented in which the participants were not stimulated with odor or image. In total, nine conditions were presented (Figure 1b): a blank condition without odor and picture; four unimodal conditions with only odor or only visual stimuli, and both pleasant or unpleasant; two bimodal conditions with congruent stimulus combinations (either pleasant or unpleasant),for example, odor of a rose and image of a rose; and two bimodal conditions with incongruent stimuli, for example, odor of dirty socks and image of a rose and vice versa.

The auditory paradigm was conducted in the same manner as the olfactory paradigm.

The presentation of the stimuli during both fMRI tasks and the recording of the subjects' feedback and scanner triggers were achieved using the software E‐Prime 2.0 (Schneider, Engel, & Debener, 2008). The presentation was projected onto an MRI‐compatible screen, visible via a mirror mounted to the head coil. In addition to oral instructions prior to the measurement, all participants read the instructions on the screen in the MRI scanner.

Figure 1a illustrates the experimental procedure. The order of conditions was pseudorandomized with each condition being repeated eight times, resulting in a total of 88 trials. Each trial started with a black crosshair for 1,760–6,160 ms (mean 3,960 ms, *SD* 2200 ms), during which time the subjects had to fixate the crosshair. A color switch to green (range of 1,400–1800 ms, mean duration = 1,600 ms, *SD* = 200 ms) indicated preparation for stimulus arrival. To provide simultaneous odor and visual stimulation and to control for the delay of the odor stimulation, the image was presented 700 ms after the odor. For the auditory paradigm, sound and images were turned on simultaneously. Each image was presented for 1.5 s, followed by a second black fixation crosshair of 2 s duration. At the end of each trial, the participants rated the pleasantness of either the odor/the sound or the image on a 10‐point Likert scale, using the LUMItouch response system (LUMItouch; Photon Control, Burnaby, Canada), a button press device held in the right hand. The index and ring fingers were used to scroll from 1 = unpleasant to 10 = pleasant and the middle finger to confirm the entry. The visual stimuli were presented with either a yellow (no simultaneous odor) or a blue fixation cross (simultaneous odor), which the participants had learned to decode prior to the measurement. The use of these color‐coded fixation crosses as an indicator of the presence of an odor prevents random activity within the olfactory cortex which may be caused by attention modulation (Zelano et al., 2005).

### 2.5 Acquisition of fMRI data

Neuroimaging data were acquired using a 3T Prisma MR scanner (Siemens Medical Systems, Erlangen, Germany) located in the Medical Faculty of RWTH Aachen University. Functional images were collected with an echo‐planar imaging (EPI) T2* weighted contrast sequence sensitive to blood‐oxygen‐level‐dependent (BOLD) contrast (voxel size: 3 × 3 × 3 mm^3^, 64 × 64 matrix, field of view: 192 × 192 mm^2^, 34 slices, whole‐brain acquisition, interleaved, no spacing between slices, repitition time (TR) = 2 s, echo time (TE) = 28 ms, alpha = 77°). The EPI sequences were 18 min long on average with approximately 540 scans.

High‐resolution T1‐weighted structural images were acquired by means of a three‐dimensional magnetization‐prepared rapid acquisition with gradient echo (MPRAGE) sequence (voxel size: 1 × 1 × 1 mm^3^, sagittal FoV: 256 × 256 mm^2^, 160 slices, TR = 1.9 s, TE = 2,520 ms, alpha = 9°). The duration of the MPRAGE sequence was 4:35 min.

### 2.6 Analysis of behavioral data

Pleasantness ratings of odors, sounds, and photographs were collected during the fMRI experiment. For each condition (unimodal and bimodal), the mean pleasantness rating was calculated and used as a dependent variable.

The behavioral data were analyzed using SPSS 23 (IBM Statistics). For a three‐way group × pleasantness × congruency repeated‐measures analysis of variance, items were assigned to each level of the two within‐subject factors task (pleasant or unpleasant) and congruency (congruent or incongruent) and the between‐subjects factor group (ASD and HC).

### 2.7 fMRI data analysis

Images were analyzed using the Statistical Parametric Mapping 12 (SPM12) software implemented in MATLAB 2015b (MathWorks, Inc., Natick, MA). Due to T1 stabilization effects, the first four images of each time series were discarded. Preprocessing of the fMRI data included the adjustment of the origin of all images to the anterior commissure before slice timing to provide better normalization to Montreal Neurological Institute (MNI) space. Slice timing temporally corrected the acquisition to the middle image slice (Slice 18) with the TR of an interleaved ascending (bottom‐middle) acquisition order. During realignment, functional scans were spatially corrected for individual head movements and were subsequently coregistered to the anatomical scan. The anatomical image was segmented in gray matter, white matter, and cerebrospinal fluid with the help of tissue probability maps, and the estimates of spatial normalization parameters in the MNI standard space were calculated (voxel size = 3 × 3 × 3 mm^3^). These parameters were applied to the functional images and used in the normalization step, following which the normalized EPI data were spatially smoothed with an isotropic Gaussian kernel (full‐width at half‐maximum  =  8 mm). All coordinates are in reference to the MNI convention (http://www.mni.mcgill.ca).

For each subject, delta functions with the time points of each type of trial presentation were convolved with the canonical hemodynamic response function (HRF) to build a regression model of the time series. Realignment parameters of each participant were included as nuisance variables. Additional HRF‐convolved regressors of no interest were the onsets of the rating scales. A high‐pass filter with a cutoff period of 128 s was applied and serial autocorrelations were accounted for by including a first‐order autoregressive covariance structure (AR(1)).

An SPM12 random‐effects analysis was performed by entering all conditions into a full factorial design. The statistical thresholds of the general linear models (GLM), unless otherwise mentioned, was set at *p* < .05 corrected for multiple comparisons at the voxel level using Gaussian random field theory as implemented in SPM (familywise error correction [FWE]). For the analysis of the odor–visual as well as audio–visual integration, we used the “superadditivity” index by Stevenson et al. (2014): (AV > A + V) or (OV > O + V).

### 2.8 Dynamic causal modeling

DCM is a mathematical framework used to estimate effective connectivity that one neural system exerts over another and how this coupling is influenced by changes in the experimental context (Friston, 2005; Friston, Harrison, & Penny, 2003). Here, we used DCM (DCM12) to assess the cortical information exchange underlying multisensory (olfactory–visual as well as auditory–visual) stimulation.

Focusing on the regions identified in unimodal and bimodal stimulation, our analysis revealed how activity in the IPS gates the reciprocal connections between the primary sensory regions. During unimodal stimulation, relevant regions of interest (ROIs) were selected based on activation peaks in visual, olfactory as well as auditory stimulation (MNI coordinates: visual: cuneus = 18/−100/14, auditory: superior temporal gyrus (STG) = 60/−16/8, olfactory: amygdala = 24/−1/−16). To investigate the effective connectivity of the IPS with the visual and auditory (AV) and visual and olfactory (OV) cortices during MSI, relevant activation peaks of the respective contrasts of paradigms were selected (IPS_AV_ = −27/−55/47, IPS_OV_ = −27/−52/50). Individual subject‐level peaks were identified within a 9 mm search volume around the group level peaks for each of the contrasts. Next, we extracted the average eigenvariate time series from a 6 mm sphere around the individual peaks, adjusted by the effect of interests across the relevant session.

**Figure 2:**
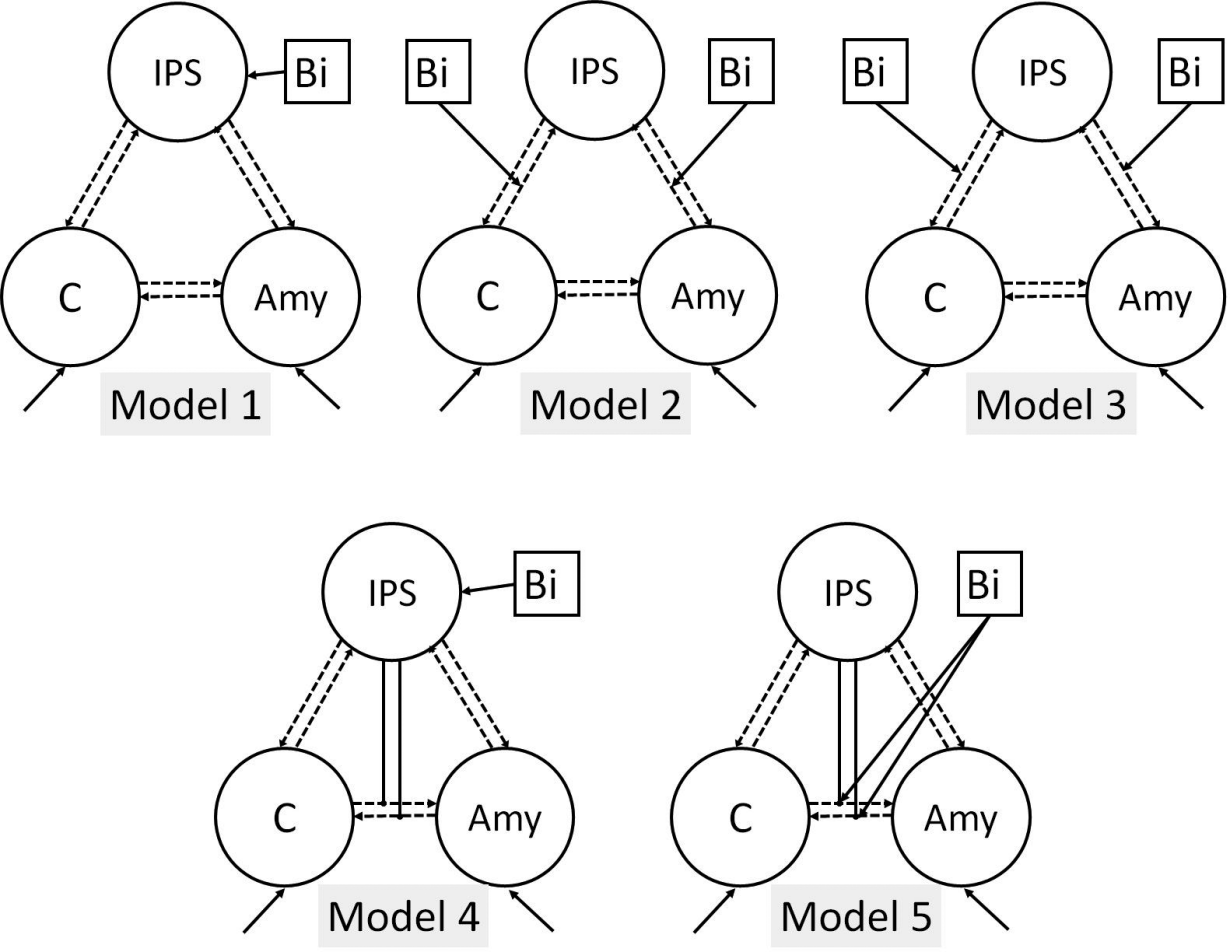
DCM model space for the olfactory–visual integration. The model space for auditory–visual integration was identical, with the amygdala being replaced by the SG. Endogenous, modulatory, and direct effects as well as nonlinear modulatory effect (A‐, B‐, C‐, and D‐matrix, respectively) are shown. Self‐connections are not depicted in the model space. Amy, amygdala; bi = bimodal stimulation; C, cuneus; DCM, dynamic causal modeling; IPS, inferior parietal sulcus; STG, superior temporal gyrus

Five different deterministic models (Figure 2), including linear as well as nonlinear effects, were created (one state per region). The same full endogenous connectivity pattern including self‐connections (A‐matrix) was assumed for all five models, with bilateral fixed connections between all three regions during both sessions. Driving inputs (audio–visual events, olfactory–visual events) were set to modulate neuronal activity in the visual, auditory, and olfactory cortices, respectively, with the bimodal stimulation being modeled as a direct or modulatory input. The structures of the models in both model spaces were the same with respect to the driving and modulatory effects as well as the connectivity structure of the nodes. The only difference between the model spaces yet in analogy to each other was one of the three nodes representing a sensory brain region of the respective stimulated sensory modality in each task: for the audio–visual task, this region was the STG whereas the amygdala was selected for the olfactory–visual task.

Models were specified as follows: the influence of bimodal stimulation was included as either driving input (C‐matrix) on the IPS (Models 1 and 4) or as a modulatory input (B‐matrix) on the reciprocal connections between (a) amygdala ↔ IPS and cuneus ↔ IPS, for the olfactory–visual session, and between STG ↔ IPS and cuneus ↔ IPS, for the auditory–visual session (Models 2 and 3), or (b) amygdala ↔ cuneus for the olfactory–visual session, and STG ↔ cuneus for the auditory–visual session (Model 5). The nonlinear modulatory effects (D‐matrix) were modeled as top‐down influence from the IPS on the connection between amygdala ↔ cuneus, for the olfactory–visual session, or STG ↔ cuneus, for the auditory–visual session (Models 4 and 5).

Random‐effects Bayesian model selection (BMS), which accounts for between‐subjects heterogeneity, was used to identify the structure of the model(s) with the highest evidence based on the data for each session and for each group. This random effects approach has been regarded as the method of choice for clinical studies (Stephan, Penny, Daunizeau, Moran, & Friston, 2009). Results are reported in terms of expected posteriors and exceedance probabilities, with the latter indicating the likelihood of one specific model compared to any other in the comparison set (Stephan et al., 2009).

## 3 RESULTS

### 3.1 Behavioral data

No effects of group or group interaction with either the pleasantness conditions or the congruency conditions were found in either the olfactory–visual or the auditory–visual paradigm. A detailed description of the behavioral results is provided in the Supporting Information.

### 3.2 fMRI data

#### 3.2.1 Effect of unimodal stimulation

As no significant group effect was observed in either session, the following analyses were collapsed across the groups.

**Figure 3:**
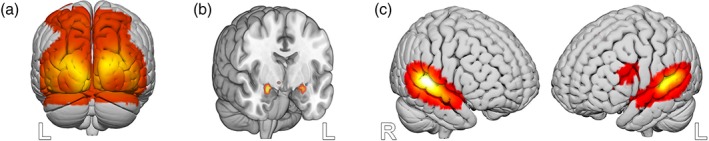
(a). Visual stimulation. Activation is depicted at *p* < .05 FWE corrected at the voxel level. (b) Olfactory stimulation. Brain activation is presented at an uncorrected threshold of *p* < .001 for visualization purposes. (c) Auditory stimulation. Activation is depicted at *p* < .05 FWE corrected at the voxel level. FWE, familywise error [Color figure can be viewed at http://wileyonlinelibrary.com]

In response to visual stimulation (Figure 3a), significant hemodynamic activation was found in a widespread cluster (13,962 voxels) with a peak level activation in the left middle occipital gyrus (MNI: −15/−97/5, *T* = 26.16). The cluster also extended to the right cuneus, the bilateral calcarine and the bilateral fusiform gyri, the superior parietal gyrus, and the medial frontal gyrus (MFG). Increased activation was also found in the bilateral inferior frontal gyrus (IFG; left: peak MNI: −42/47/−10, *T* = 3.36, 15 voxel; right: peak MNI: 42/11/29, *T* = 4.85, 475 voxels), the right insula (peak MNI: 33/−23/−1, *T* = 25.96, 86 voxels), and the right thalamus (peak MNI: 12/−1/2, *T* = 3.20, five voxels).

In response to olfactory stimulation (Figure 3b), an increased BOLD response was observed in the bilateral amygdala (right: peak MNI: 24/−1/−16, *T* = 6.61, 26 voxels; left: peak MNI: −21/−4/−13, *T* = 5.26, five voxels), with the cluster located in the right amygdala extending to the right hippocampus.

In response to auditory stimulation (Figure 3c), there was increased activation in the bilateral STG (right: peak MNI: 60/−16/8, *T* = 19.38, 984 voxels; left: peak MNI: −51/−22/8, *T* = 18.06, 904 voxels) including the bilateral Heschl's gyrus. The left IFG (p. triangularis; peak MNI: −45/17/26, *T* = 5.21, 79 voxels) and the left MFG (peak MNI: −6/17/44, *T* = 5.39, 42 voxels) were also triggered by auditory stimulation.

#### 3.2.2 Effect of bimodal stimulation

No significant group effects, or group × session interaction effect or group × congruency interaction effect, were seen in either session. The following analyses were therefore collapsed across the groups. Additionally, no significant suprathreshold activations were found when comparing congruent > incongruent trials or vice versa. For a complete presentation of the results, the following section first shows contrasts collapsed across congruent and incongruent conditions within each session. The individual analyses (both congruent and incongruent trials) can be seen in the Supporting Information (Table S1).

**Figure 4:**
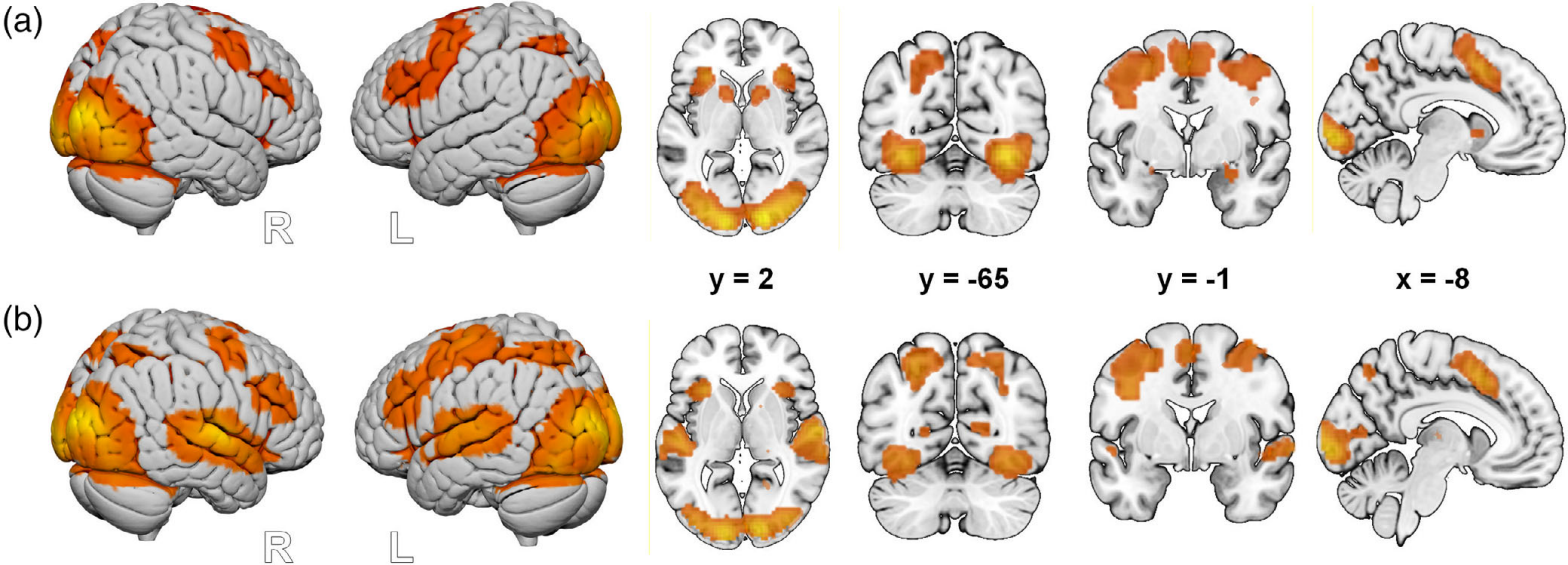
Increased neural activation of multisensory integration. (a) Activation clusters during olfactory–visual integration. (b) Activation clusters during auditory–visual integration. Activation is depicted at *p* < .05 familywise error (FWE) corrected at the voxel level [Color figure can be viewed at http://wileyonlinelibrary.com]

The olfactory–visual stimulation (OV > O + V) led, on the one hand, to a stronger involvement of areas associated with visual processing (the cuneus, and the bilateral calcarine, and fusiform gyri) and, on the other hand, to regions associated with olfactory stimulation (in particular, the right amygdala/hippocampus region) (Figure 4a, Table 2). Stronger activation was observed also in the precentral gyrus, the bilateral IFG/MFG, the bilateral supplementary motor area (extending to midcingulate cortex) and the bilateral anterior insula.

**Table 2:** MNI coordinates of significant clusters of olfactory–visual and auditory–visual stimulation across both groups and across incongruent and congruent trials

Anatomical region	Side	k	Peak voxel			
			T	x	y	z
Olfactory–visual integration (OV > O + V)						
Fusiform gyrus	R	3,807	16.72	27	−79	−7
Calcarine gyrus	R		16.63	18	−94	5
Cuneus	R		16.14	21	−97	11
Calcarine gyrus	L		15.35	−12	−94	−1
Fusiform gyrus	R		14.48	33	−58	−10
Fusiform gyrus	L		13.04	−33	−52	−13
Fusiform gyrus	L		12.68	−30	−58	−10
Superior parietal lobule (extending to IPS)	L		7.35	−27	−52	50
Precentral gyrus	L	629	9.33	−30	−7	50
IFG (p. opercularis)			7.32	−39	5	29
Middle frontal gyrus			5.23	−48	26	32
Posterior medial frontal cortex	L	609	10.59	−6	8	50
Midcingulate cortex	R		9.84	9	11	44
IFG (p. opercularis)	R	171	7.35	42	8	26
Insula lobe	L	165	9.36	−30	23	2
IFG (p. opercularis)			4.90	−24	29	−13
Middle frontal gyrus	R	158	6.71	30	−4	53
Insula	R	132	8.20	30	23	2
Caudate (extending to putamen)	R	51	6.47	12	8	2
Putamen (extending to caudate)	L	38	6.15	−12	8	2
Amygdala	R	28	6.13	24	−1	−16
Hippocampus			6.07	21	−4	−13
Thalamus	R	6	4.89	9	−16	8
Amygdala	L	6	5.11	−27	−7	−13
IFG (p. orbitalis)	R	4	5.15	24	29	−13
Auditory–visual integration (AV > A + V)						
Cuneus	R	4,498	12.15	18	−100	14
Middle occipital gyrus	R		11.26	30	−91	14
Calcarine gyrus	L		11.24	−12	−94	−1
Middle occipital gyrus	L		11.15	−18	−97	8
Lingual gyrus	L		10.65	−24	−79	−13
Fusiform gyrus	R		10.31	27	−79	−7
Fusiform gyrus	L		9.87	−33	−49	−16
Fusiform gyrus	R		9.50	30	−49	−16
Postcentral gyrus	L		8.68	−36	−22	53
Inferior parietal gyrus (extending to IPS)	L		8.06	−27	−55	47
Superior parietal gyrus (extending to IPS)	L		8.04	−27	−61	50
STG	R	628	11.15	63	−16	8
Temporal pole			7.08	60	5	−7
STG	L	571	9.62	−51	−25	8
Posterior medial frontal cortex	L	436	9.81	−3	11	50
Posterior medial frontal cortex	L		9.75	−6	14	47
Posterior medial frontal cortex	R		9.09	6	14	47
Insula lobe	L	119	7.98	−30	23	2
IFG (pars opercularis)	R	100	6.29	42	8	29
Precentral gyrus	R	97	6.07	30	−7	50
Middle frontal gyrus			5.86	39	−1	62
Insula lobe	R	74	6.17	33	23	−1
Calcarine gyrus	R	40	5.32	18	−64	8
Thalamus	L	11	5.04	−9	−19	8
Postcentral gyrus	R	8	4.84	48	−31	47
IFG (pars triangularis)	R	6	4.76	51	32	20
Thalamus	R	5	4.89	21	−28	−1

*Note*. Local maxima of activated clusters shown at *p* < .05 FWE corrected at the voxel level.

Abbreviations: FWE, familywise error; IFG, inferior frontal gyrus; IPS, inferior parietal sulcus; MNI, Montreal Neurological Institute; STG, superior temporal gyrus.

The auditory–visual stimulation (AV > A + V) was linked to increased neural activation in the visual areas as well as the areas associated with auditory processing (the bilateral superior temporal gyri). Other clusters with significant stimulation were found in the precentral gyrus, the bilateral IFG/MFG, the bilateral anterior insula, the bilateral thalamus, and the supplementary motor area (Figure 4b, Table 2).

#### 3.2.3 Amodal MSI

**Figure 5:**
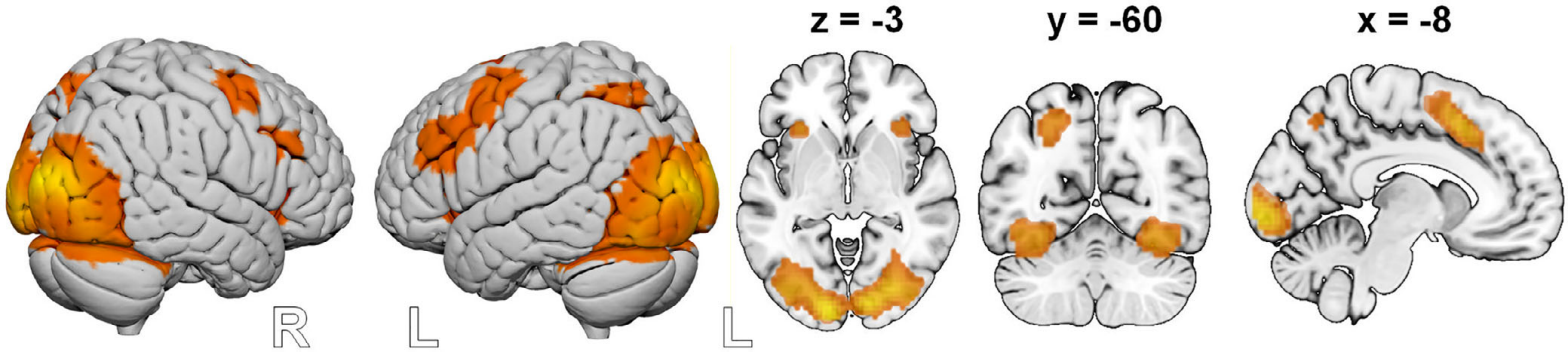
Areas recruited during multisensory integration. Results are based on a conjunction including both paradigms (AV > A + V ∩ OV > O + V). Activation is depicted at *p* < .05 familywise error (FWE) corrected at the voxel level [Color figure can be viewed at http://wileyonlinelibrary.com]

To isolate the brain activity linked to general amodal MSI, we performed a conjunction analysis across both sessions. The analysis revealed activation in areas associated with visual processing (the cuneus, the middle/inferior occipital gyrus, the calcarine gyrus, the fusiform gyrus, and the precuneus) and in the parietal areas linked to MSI (the superior parietal gyrus and the IPS). Other clusters were found in the middle frontal/precentral gyrus, the supplementary motor area (extending to the midcingulate cortex), the bilateral insula, and the bilateral IFG (p. opercularis) (Table 3, Figure 5).

**Table 3:** MNI coordinates of peak voxel within significant clusters of the conjunction analyses (AV > A + V ∩ OV > O + V across both groups

Anatomical region	Side	K	Peak voxel			
			T	x	Y	Z
Cuneus	R	3,034	12.15	18	−100	14
Middle occipital gyrus	R		11.26	30	−91	14
Calcarine gyrus	L		11.24	−12	−94	−1
Middle occipital gyrus	L		11.15	−18	−97	8
Lingual gyrus	L		10.65	−24	−79	−13
Fusiform gyrus	R		10.31	27	−79	−7
Fusiform gyrus	L		9.87	−33	−49	−16
Fusiform gyrus	R		9.50	30	−49	−16
Inferior parietal lobule (extending to IPS)	L		7.31	−27	−52	47
Superior parietal lobule	L		6.13	−15	−67	50
Middle occipital gyrus	L		5.50	−27	−73	29
Middle frontal gyrus	L	505	8.00	−30	−4	53
Precentral gyrus			7.20	−39	2	32
Posterior medial frontal cortex	L	378	9.81	−3	11	50
Posterior medial frontal cortex	L		9.75	−6	14	47
Posterior medial frontal cortex	R		9.09	6	14	47
Insula	L	88	7.98	−30	23	2
Precentral gyrus	R	66	6.07	30	−7	50
IFG (p. opercularis)	R	64	6.29	42	8	29
Insula	R	59	6.17	33	32	−1

*Note*. Local maxima of activated clusters shown at *p* < .05 FWE corrected at the voxel level.

Abbreviations: FWE, familywise error; IFG, inferior frontal gyrus; MNI, Montreal Neurological Institute.

#### 3.2.4 Sensory‐specific MSI

Comparing olfactory–visual versus auditory–visual stimulation (OV – O – V > AV – A – V), we found greater activation in a cluster encompassing the right fusiform gyrus and the right lingual gyrus (peak MNI: 30/−67/−7, *T* = 5.54, 86 voxels) and the right amygdala (peak MNI: 24/−4/−13, *T* = 4.65, three voxels).

The reverse contrast (auditory–visual > olfactory–visual; AV – A – V > OV – O – V) revealed greater neural activation in the bilateral STG (left: peak MNI: −54/−22/8, *T* = 8.64, 408 voxels; right: peak MNI: 63/−16/8, *T* = 9.45, 389 voxels).

No significant group differences were seen in either contrast.

#### 3.2.5 Dynamic causal modeling

**Figure 6:**
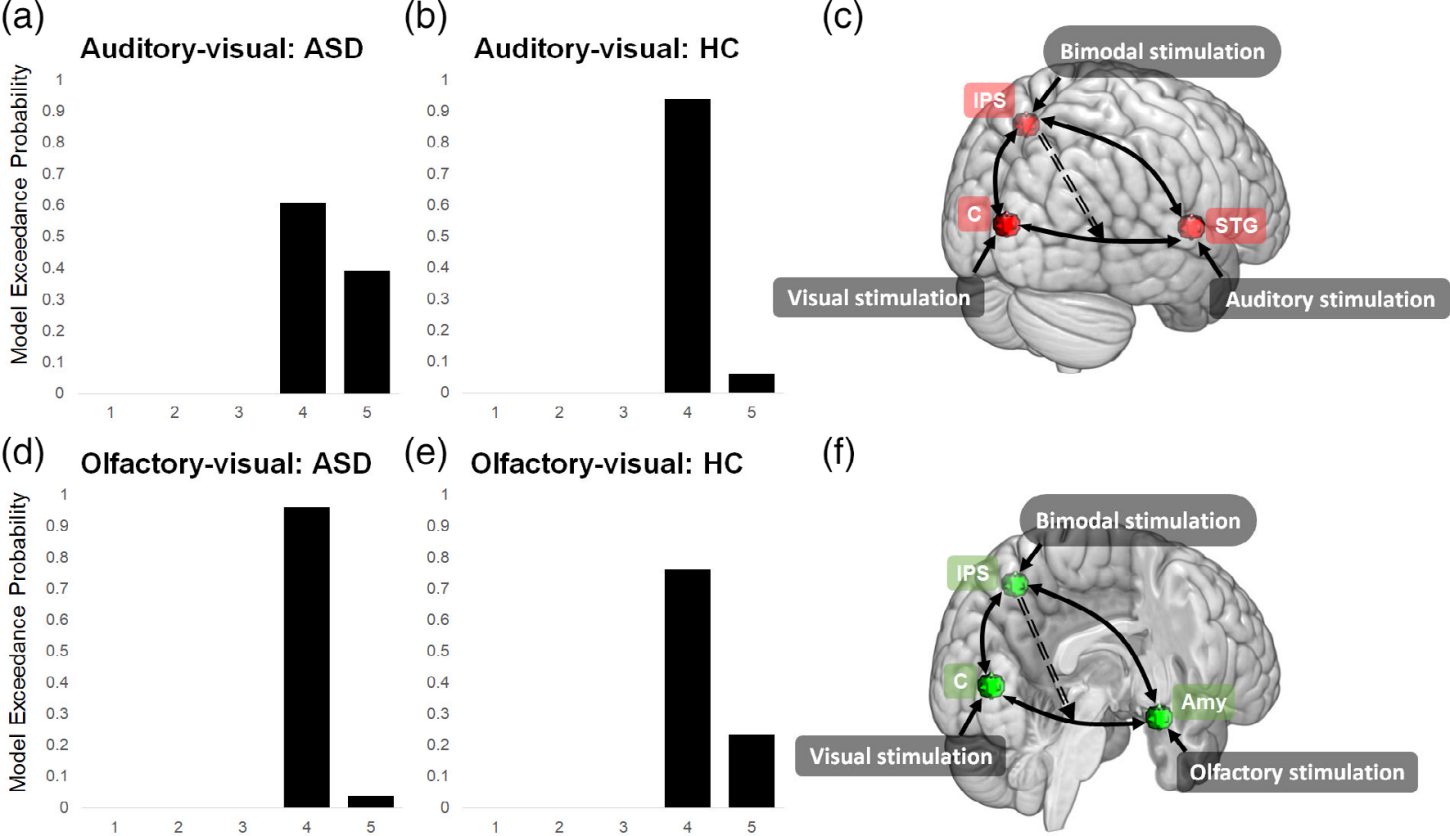
Model exceedance probabilities for Models 4 and 5, during auditory–visual integration in (a) patients with ASD, and (b) HCs, and during olfactory visual integration in (d) patients with ASD and (e) HCs. (c) Effective connectivity pattern for auditory–visual stimulation. (f) Effective connectivity pattern for olfactory–visual stimulation. Amy, amygdala; ASD, autism spectrum disorder; bi, bimodal stimulation; C, cuneus; HC, healthy control; IPS, inferior parietal sulcus; STG, superior temporal gyrus [Color figure can be viewed at http://wileyonlinelibrary.com]

Following the inversion of five alternative DCMs per subject and per experiment, random effects BMS showed that, in both groups and during both olfactory–visual and auditory–visual integration, Models 4 and 5 clearly outperformed all other models (see Figure 6 and Table 4). This indicates that the nonlinear models in the model space were definitely more supported by the data than the linear ones, which was corroborated by a random‐effects inference on the family level (see Figure S2, Supporting Information). Moreover, Model 4 was always the winning model with expected posteriors of more than 40% for both conditions and both groups. Model 5 always had an expected posterior of more than 20% (see Table 4), indicating an additional modulatory effect of the multisensory input on the connection between the cuneus and the respective modality‐specific region. Expected posteriors of all other models were lower than 10%, mostly not even exceeding 5 (see Table 4). Model 4 was composed of a driving input of bimodal stimulation (Bi) directly on IPS, and (nonlinear) modulations from IPS on the reciprocal connections between the cuneus ↔ amygdala (olfactory–visual stimulation) and the cuneus ↔ STG (auditory–visual stimulation). Model 5 also included the top‐down effect of the IPS on the connection between the cuneus and the respective modality‐specific region (amygdala or STG), but, compared to Model 4, a direct input of bimodal stimulation to the IPS was omitted and instead a modulatory effect of bimodal stimulation on the above‐mentioned reciprocal connection was included. More detailed information about subject‐specific model preferences is provided in the Supporting Information (see Table S2), including the free energy of all models and the models in Occam's window for all subjects.

**Table 4:** Expected probability of each model across both experiments and both groups

	Expected probability				
	Model 1	Model 2	Model 3	Model 4	Model 5
Olfactory–visual integration					
ASD	.046	.05	.048	.613	.242
HCs	.072	.048	.048	.493	.34
Auditory–visual integration					
ASD	.044	.044	.044	.461	.407
HCs	.045	.046	.046	.594	.269

Abbreviations: ASD, autism spectrum disorder; HC, healthy control.

Because random‐effects analyses on the coupling parameters of a DCM rest on the assumption that one single model generated the parameters, we decided to perform‐dependent *t* tests twice, namely, on the parameters of the winning Model 4 and on those of Model 5. The rationale for this approach was the following: if the tests on the parameters of these two models yielded concordant results, they would reflect some noteworthy information about the “true” coupling of the regions, since the sum of the expected posteriors of these two models easily exceeded 80%. Based on this, during the auditory–visual stimulation, significantly higher coupling parameters were seen from the cuneus > STG (*M* = 0.17, *SD* = .24) compared to the opposite direction (STG > cuneus, *M* = −0.02 (*SD* = .22); *t*(34) = 3.11, *p* = .004). Likewise, for Model 5, we observed substantially higher coupling parameters from the cuneus > STG (*M* = 0.25, *SD* = .28) compared to the opposite direction (STG > cuneus, *M* = 0.008 (*SD* = .24); *t*(34) = 3.11, *p* = .002). During the olfactory–visual stimulation, coupling parameter of cuneus > amygdala (Model 4: *M* = .07, *SD* = .13, Model 5: *M* = .098, *SD* = .26), and amygdala ‐ > cuneus (Model 4: *M* = −.003, *SD* = .26, Model 5: *M* = .019, *SD* = .27) did not differ significantly.

## 4 DISCUSSION

This study compared two independent cross‐modal paradigms delineating the specifics of as well as the commonalities between the networks linked to the integration of visual information and either olfactory or auditory input in healthy participants as well as in patients with ASD.

### 4.1 Network related to multimodal integration

Using a conjunction analysis, we sought to determine the neuronal circuitries involved in multimodal integration irrespective of the type of sensory stimuli, and identified a network of brain areas spanning the prefrontal, the parietal, and the visual cortices. Thus, during MSI irrespective of the sensory stimuli (but always including visual information), we observed a conjoint recruitment of the brain network related to visual processing (the striate and extrastriate cortices), spatial perception and sustained attention (the parietal cortex) (e.g., Malhotra, Coulthard, & Husain, 2009), inhibition and multisensory attention (the insula) (e.g., Ghahremani, Rastogi, & Lam, 2015), vigilance, error response monitoring and resolution of response conflict (the medial prefrontal cortex) (Chechko et al., 2009; e.g., Chechko et al., 2012; Chechko et al., 2016), and, finally, motor output (the precentral gyrus). This corroborates the findings of studies that focused on the integration of auditory stimuli with other sensory modalities and favored the role of those areas in MSI processes, often referring to the IPS as one of the central hubs (Bremmer et al., 2001; Calvert & Thesen, 2004; Grefkes & Fink, 2005). Imaging studies in humans and nonhuman primates have established the parietal cortex to be involved in the localization and attention of cues and targets (for a review, see Calvert & Thesen, 2004; Macaluso, 2006). Crucially, the spatial and temporal consistency of cross‐modal stimuli strongly influences information integration in the parietal cortex (Macaluso, 2006).

While the human capacity for optimal MSI appears to be quite pervasive, precisely how multiple sensory cues are integrated in the neural circuitry remains largely unknown. To determine the role of the IPS in the information exchange in both audio–visual and olfactory–visual integration, we searched a small model space comprising plausible connectivity patterns using the DCM and BMS. Regardless of whether olfactory or auditory information had to be integrated with visual information, we found the model structure to be very similar. The commonalities of the models reveal a driving input of bimodal stimulation to the IPS, which in turn exerts a modulatory effect on the reciprocal connections between the respective primary sensory regions (the amygdala and the visual cortex in olfactory–visual integration, or the STG and the visual cortex in audio–visual integration). With regard to audio–visual integration, our results are consistent with other evidence showing that the IPS exerts a top‐down control on the information exchange between the primary sensory regions (Regenbogen et al., 2017). Our study also corroborates the notion of the IPS playing a role in olfactory–visual integration (Gottfried & Dolan, 2003; Seubert et al., 2010), suggesting that, analogous to its role in audiovisual integration, the IPS is involved in a top‐down control for the processing of inputs from the primary sensory regions (Bressler, Tang, Sylvester, Shulman, & Corbetta, 2008; Hopfinger, Buonocore, & Mangun, 2000).

We also observed a pronounced activation in brain areas involved in different levels of visual processing during MSI (the right cuneus, the bilateral fusiform, and the middle occipital gyri) during auditory–visual integration. In addition, in the whole group, the coupling parameters for the connection from the visual cortex to the auditory cortex were higher than for the opposite connection both in the winning Model 4 and in Model 5. This indicates that the visual enhancement of other sensory processes is central to the understanding of sensory interaction, supporting the Colavita visual dominance effect (Colavita, 1974) and a resulting shift of attention toward the visual domain (Posner, Nissen, & Klein, 1976). For instance, in case of clear visual stimuli, the visual processing robustly mediates the processing of multisensory information (Regenbogen et al., 2017). Thus, our results suggest that the information exchange between the visual cortex and the auditory cortex represents an earlier processing phase of MSI, which has been already observed in audio–visual integration studies (Foxe & Schroeder, 2005; Kayser, Logothetis, & Panzeri, 2010; Lakatos, Chen, O'Connell, Mills, & Schroeder, 2007). The role of the visual areas in MSI with olfaction had already been demonstrated, indicating the need of information exchange between the olfactory and visual areas to facilitate integration (Lundström et al., 2018; Ripp et al., 2018). In our experiment, the coupling parameters for the connection from the visual cortex to the olfactory cortex did not differ significantly from the opposite connection either in the winning Model 4 or in Model 5. The amygdala belongs to an affective circuit that generates a psychological state of pleasantness and unpleasantness and enhances the visual processing of the target object (Amaral et al., 2003; Barrett et al., 2007). Pessoa et al. (2006) suggest that activation of the amygdala may depend on visual perception, since the affective state may dictate target visibility. In this context, we suggest that the amygdala may have the same strength of modulatory control over the visual system as vice versa.

The role of the insula has been elucidated during the processing of simultaneously presented deviant auditory and visual stimuli, with responses in the dorsal anterior cingulate cortex (dACC), the lateral frontal and the posterior parietal cortices having been found to be mediated by the right anterior insula (Chen et al., 2015). Also, activation patterns of the insula and the putamen have been shown to be connected during bimodal olfactory–visual stimulation, as opposed to unimodal stimulation (Ripp et al., 2018), which appear to be specific to MSI. The medial PFC, including the dACC, has been found to be involved in the top‐down modulation of sensory processing (Chen et al., 2015; Debener, Kranczioch, Herrmann, & Engel, 2002; Regenbogen, Habel, & Kellermann, 2013) and MSI, especially with respect to reorienting attention to the relevant stimuli (Orr & Weissman, 2009).

### 4.2 Effects of the type of sensory stimulation on the network related to multimodal integration

Our study not only shows commonalities among the brain networks linked to MSI, but it also demonstrates sensory‐specific integration effects. Consistent with the fMRI literature with respect to unisensory processing, we observed activation in the amygdala during olfactory processing (e.g., Seubert et al., 2013), activation of the STG as well as the IFG and the dorsomedial prefrontal cortex (dmPFC) during auditory stimulation, and activation of the striate/extrastriate visual areas, the dmPFC and the insula/IFG during visual stimulation (e.g., Costafreda, Brammer, David, & Fu, 2008; Nourski, 2017; Salomon et al., 2016). Examining the network linked to the integration of visual and olfactory information (OV > O + V), we observed activation in the amygdala, the putamen and the caudate, which dropped out at the conjunction with the respective contrast for audio–visual integration. Comparing the networks linked to the integration of visual and olfactory information, as opposed to the integration of visual and acoustic information (OV – O – V > AV – A – V), we noticed increased activation in the right fusiform gyrus and the left lingual gyrus as well as activation in the right amygdala. The observation of amygdala activation is in line with the fMRI studies in which olfactory processing has been seen to elicit activation in the orbitofrontal regions as well as the piriform cortex including the amygdala, with amygdala activation in particular having been seen to be strongly triggered by odors (Costafreda et al., 2008; Kadohisa, 2013; Seubert et al., 2013). The amygdala, which receives a direct input from the olfactory bulb (Kadohisa, 2013), is assumed to play a role in evaluative behavior and affective response within associative learning, the encoding, for instance, of positive or negative cues (Schoenbaum, Chiba, & Gallagher, 1999; Seubert et al., 2013). In addition, it is worth mentioning that the visual areas are more engaged during simultaneous olfactory stimulation as opposed to simultaneous auditory stimulation, indicating more reliance on the visual domain when integrating olfactory as compared to auditory information with visual input. Hence, according to these results, on the neural level, olfaction seems to be less influential with respect to the integration process than audition. Furthermore, during auditory–visual integration (AV > A + V), as opposed to olfactory–visual integration (AV – A – V > OV – O – V), we found increased activation in the STS/STG. Imaging studies have shown that the posterior STS/STG plays an important role in audio–visual sensory integration, involving a range of stimuli and tasks such as multisensory object recognition and audio–visual speech comprehension (Beauchamp, Argall, Bodurka, Duyn, & Martin, 2004; Beauchamp, Lee, et al., 2004; Calvert, Hansen, Iversen, & Brammer, 2001; Stevenson, Geoghegan, & James, 2007; Weisberg, Hubbard, & Emmorey, 2017).

### 4.3 Intact MSI in adults with ASD

The ability to integrate multisensory information develops during ontogeny with an individual's progressive exposure to the environment's versatile stimulation (Stein et al., 2014). Adequate experience in terms of exposure to the environmental statistical regularities is required to identify multiple sensory inputs from different modalities and to fully benefit from multisensory cues that develop over time (Gori, Del Viva, Sandini, & Burr, 2008; Nardini, Bedford, & Mareschal, 2010; Stein et al., 2014). This applies to both typically developing individuals (Burr & Gori, 2012; Gori et al., 2008; Nardini et al., 2010) and patients with ASD (Beker et al., 2018; Brandwein et al., 2013; Foxe et al., 2015). As a developmental disorder, ASD is quite heterogeneous and nonstatic, contrary to the common and quite oversimplified perception that autistic behavior is stable over time. Thus, several studies have demonstrated that, especially in adults with Asperger syndrome, capacities pertaining to improvements in executive function (Weiss et al., 2018) as well as theory‐of‐mind abilities and empathy develop over time (Pellicano, 2010). These changes are attributed to brain maturation on the one hand (Selemon, 2013) and, on the other, to the development of compensatory mechanisms (Schulte‐Rüther et al., 2014). According to Schulte‐Rüther et al. (2014), early behavioral therapeutic interventions contribute to developmental effects in the neural networks, leading to the formation of neural compensatory strategies such as more controlled processing or greater neural supervisory schemes.

In the present study, no differences in MSI processing were found between HCs and patients with ASD, which is consistent with the growing evidence of amelioration of MSI deficits in individuals with ASD during adolescence (Beker et al., 2018; Foxe et al., 2015). Measurements of audio–visual integration have shown children with ASD to be able to catch up with their typically developing counterparts at later stages of development (Foxe et al., 2015; Stevenson, Siemann, et al., 2014; Taylor et al., 2010). It should be pointed out, however, that the individuals examined in the present study were adults with largely normal‐range IQs. Thus, the observation that our sample of ASD patients was on par with the typically developing control group may be attributed to the specific properties of the ASD sample, which was not representative of the whole spectrum of the disorder.

### 4.4 Summary

Taken together, the results of the present study show an overlapping network of brain regions involved in MSI of both olfactory–visual and audio–visual information in both patients with ASD and healthy individuals. The results also demonstrate an enhanced recruitment of the IPS to modulate changes between areas relevant to sensory perception across the sensory modalities. However, whether adults with ASD employ compensatory mechanisms to experience MSI, and benefit specifically from the visual enhancement of other sensory cues, remain open questions. To elucidate the potential role of compensatory neural strategies, future research should keep sight of age‐related effects and behavioral therapeutic interventions in children or adolescents with ASD in order to track changes in the sensitivity of neural networks.

## CONFLICT OF INTEREST

None.

## DATA AVAILABILITY

The data that support the findings of this study are available on request from the corresponding author. The data are not publicly available due to privacy or ethical restrictions.

## Supporting information


**Figure S1** Mean pleasantness ratings and standard errors (error bar) across both groups and both experiment.
**Figure S2**. Family‐level inference with linear and nonlinear models, across both groups and both experiments.
**Table S1** Means and standard deviation of the pleasantness rating in unimodal and bimodal combination for both tasks (olfactory–visual and auditory–visual stimulation).
**Table S2** Free energy of all models across all subjects and both experiments, and model in Occam's window for all subjects.

## References

[hbm24715-bib-1003] Amaral, D. , Behniea, H. , & Kelly, J. (2003). Topographic organization of projections from the amygdala to the visual cortex in the macaque monkey. Neuroscience, 118, 1099–1120. 12732254 10.1016/s0306-4522(02)01001-1

[hbm24715-bib-0001] American Psychiatric Association . (2013). Diagnostic and statistical manual of mental disorders (5th ed.). Washington, DC: American Psychiatric Association.

[hbm24715-bib-0002] Aschenbrenner, S. , Tucha, O. , & Lange, K. W. (2000). Regensburger Wortflüssigkeits‐Test. Göttingen, Germany: Hogrefe.

[hbm24715-bib-0003] Ashwin, C. , Chapman, E. , Howells, J. , Rhydderch, D. , Walker, I. , & Baron‐Cohen, S. (2014). Enhanced olfactory sensitivity in autism spectrum conditions. Molecular Autism, 5, 53. 25908951 10.1186/2040-2392-5-53 PMC4407326

[hbm24715-bib-0004] Atanasova, B. , Graux, J. , El Hage, W. , Hommet, C. , Camus, V. , & Belzung, C. (2008). Olfaction: A potential cognitive marker of psychiatric disorders. Neuroscience and Biobehavioral Reviews, 32, 1315–1325. 18555528 10.1016/j.neubiorev.2008.05.003

[hbm24715-bib-0005] Attems, J. , Walker, L. , & Jellinger, K. A. (2014). Olfactory bulb involvement in neurodegenerative diseases. Acta Neuropathologica, 127, 459–475. http://link.springer.com/10.1007/s00401-014-1261-7 24554308 10.1007/s00401-014-1261-7

[hbm24715-bib-1004] Barrett, L. F. , Mesquita, B. , Ochsner, K. N. , & Gross, J. J. (2007). The experience of emotion. Annu Rev Psychol, 58, 373–403. 17002554 10.1146/annurev.psych.58.110405.085709 PMC1934613

[hbm24715-bib-0006] Baron‐Cohen, S. , Wheelwright, S. , Skinner, R. , Martin, J. , & Clubley, E. (2001). The autism‐spectrum quotient (AQ): Evidence from Asperger syndrome/high‐functioning autism, males and females, scientists and mathematicians (Deutsche Übersetzung von G. Dammann, 2002). Journal of Autism and Developmental Disorders, 31, 5–17. 11439754 10.1023/a:1005653411471

[hbm24715-bib-0007] Beauchamp, M. S. , Argall, B. D. , Bodurka, J. , Duyn, J. H. , & Martin, A. (2004). Unraveling multisensory integration: Patchy organization within human STS multisensory cortex. Nature Neuroscience, 7, 1190–1192. http://www.ncbi.nlm.nih.gov/pubmed/15475952 15475952 10.1038/nn1333

[hbm24715-bib-0008] Beauchamp, M. S. , Lee, K. E. , Argall, B. D. , & Martin, A. (2004). Integration of auditory and visual information about objects in superior temporal sulcus. Neuron, 41, 809–823. http://www.ncbi.nlm.nih.gov/pubmed/15003179 15003179 10.1016/s0896-6273(04)00070-4

[hbm24715-bib-0009] Beker, S. , Foxe, J. J. , & Molholm, S. (2018). Ripe for solution: Delayed development of multisensory processing in autism and its remediation. Neuroscience and Biobehavioral Reviews, 84, 182–192. 10.1016/j.neubiorev.2017.11.008 29162518 PMC6389331

[hbm24715-bib-0010] Bennetto, L. , Kuschner, E. S. , & Hyman, S. L. (2007). Olfaction and taste processing in autism. Biological Psychiatry, 62, 1015–1021. 17572391 10.1016/j.biopsych.2007.04.019 PMC2063511

[hbm24715-bib-0011] Bertone, A. , Mottron, L. , Jelenic, P. , & Faubert, J. (2003). Motion perception in autism: A “complex” issue. Journal of Cognitive Neuroscience, 15, 218–225. http://www.ncbi.nlm.nih.gov/pubmed/12676059 12676059 10.1162/089892903321208150

[hbm24715-bib-0012] Bertone, A. , Mottron, L. , Jelenic, P. , & Faubert, J. (2005). Enhanced and diminished visuo‐spatial information processing in autism depends on stimulus complexity. Brain, 128, 2430–2441. 15958508 10.1093/brain/awh561

[hbm24715-bib-0013] Brandwein, A. B. , Foxe, J. J. , Butler, J. S. , Russo, N. N. , Altschuler, T. S. , Gomes, H. , & Molholm, S. (2013). The development of multisensory integration in high‐functioning autism: High‐density electrical mapping and psychophysical measures reveal impairments in the processing of audiovisual inputs. Cerebral Cortex, 23, 1329–1341. 22628458 10.1093/cercor/bhs109 PMC3643715

[hbm24715-bib-0014] Bremmer, F. , Schlack, A. , Shah, N. J. , Zafiris, O. , Kubischik, M. , Hoffmann, K. , … Fink, G. R. (2001). Polymodal motion processing in posterior parietal and premotor cortex: A human fMRI study strongly implies equivalencies between humans and monkeys. Neuron, 29, 287–296. http://www.ncbi.nlm.nih.gov/pubmed/11182099 11182099 10.1016/s0896-6273(01)00198-2

[hbm24715-bib-0015] Bressler, S. L. , Tang, W. , Sylvester, C. M. , Shulman, G. L. , & Corbetta, M. (2008). Top‐down control of human visual cortex by frontal and parietal cortex in anticipatory visual spatial attention. The Journal of Neuroscience, 28, 10056–10061. http://www.jneurosci.org/cgi/doi/10.1523/JNEUROSCI.1776-08.2008 18829963 10.1523/JNEUROSCI.1776-08.2008 PMC2583122

[hbm24715-bib-0016] Burón, E. , Bulbena, A. , & Bulbena‐Cabré, A. (2015). Olfactory functioning in panic disorder. Journal of Affective Disorders, 175, 292–298. 25661394 10.1016/j.jad.2015.01.049

[hbm24715-bib-0017] Burr, D. , & Gori, M. (2012). Multisensory integration develops late in humans In The neural bases of multisensory processes. Boca Raton, FL: CRC Press/Taylor & Francis http://www.ncbi.nlm.nih.gov/pubmed/22593886 22593886

[hbm24715-bib-0018] Calvert, G. A. , Hansen, P. C. , Iversen, S. D. , & Brammer, M. J. (2001). Detection of audio‐visual integration sites in humans by application of electrophysiological criteria to the BOLD effect. NeuroImage, 14, 427–438. http://www.ncbi.nlm.nih.gov/pubmed/11467916 11467916 10.1006/nimg.2001.0812

[hbm24715-bib-0019] Calvert, G. A. , & Thesen, T. (2004). Multisensory integration: Methodological approaches and emerging principles in the human brain. The Journal of Physiology, 98, 191–205. http://linkinghub.elsevier.com/retrieve/pii/S0928425704000804 10.1016/j.jphysparis.2004.03.018 15477032

[hbm24715-bib-0020] Chechko, N. , Kellermann, T. , Augustin, M. , Zvyagintsev, M. , Schneider, F. , & Habel, U. (2016). Disorder‐specific characteristics of borderline personality disorder with co‐occurring depression and its comparison with major depression: An fMRI study with emotional interference task. NeuroImage: Clinical, 12, 517–525. 10.1016/j.nicl.2016.08.015 27672555 PMC5030331

[hbm24715-bib-0021] Chechko, N. , Kellermann, T. , Zvyagintsev, M. , Augustin, M. , Schneider, F. , & Habel, U. (2012). Brain circuitries involved in semantic interference by demands of emotional and non‐emotional distractors. PLoS One, 7, 1–9. 10.1371/journal.pone.0038155 PMC3362560 22666470

[hbm24715-bib-0022] Chechko, N. , Wehrle, R. , Erhardt, A. , Holsboer, F. , Czisch, M. , & Sämann, P. G. (2009). Unstable prefrontal response to emotional conflict and activation of lower limbic structures and brainstem in remitted panic disorder. PLoS One, 4, e5537 http://www.pubmedcentral.nih.gov/articlerender.fcgi?artid=PMC2680057 19462002 10.1371/journal.pone.0005537 PMC2680057

[hbm24715-bib-0023] Chen, T. , Michels, L. , Supekar, K. , Kochalka, J. , Ryali, S. , & Menon, V. (2015). Role of the anterior insular cortex in integrative causal signaling during multisensory auditory–visual attention. The European Journal of Neuroscience, 41, 264–274. 25352218 10.1111/ejn.12764 PMC4300257

[hbm24715-bib-0024] Colavita, F. B. (1974). Human sensory dominance. Perception & Psychophysics, 16, 409–412. http://www.springerlink.com/index/10.3758/BF03203962

[hbm24715-bib-0025] Costafreda, S. G. , Brammer, M. J. , David, A. S. , & Fu, C. H. Y. (2008). Predictors of amygdala activation during the processing of emotional stimuli: A meta‐analysis of 385 PET and fMRI studies. Brain Research Reviews, 58, 57–70. https://www.sciencedirect.com/science/article/pii/S0165017307002482?via%3Dihub 18076995 10.1016/j.brainresrev.2007.10.012

[hbm24715-bib-0026] Croy, I. , Symmank, A. , Schellong, J. , Hummel, C. , Gerber, J. , Joraschky, P. , & Hummel, T. (2014). Olfaction as a marker for depression in humans. Journal of Affective Disorders, 160, 80–86. 24445134 10.1016/j.jad.2013.12.026

[hbm24715-bib-0027] Debener, S. , Kranczioch, C. , Herrmann, C. S. , & Engel, A. K. (2002). Auditory novelty oddball allows reliable distinction of top‐down and bottom‐up processes of attention. International Journal of Psychophysiology, 46, 77–84. 12374648 10.1016/s0167-8760(02)00072-7

[hbm24715-bib-0028] Fairhall, S. L. , & Macaluso, E. (2009). Spatial attention can modulate audiovisual integration at multiple cortical and subcortical sites. The European Journal of Neuroscience, 29, 1247–1257. http://www.ncbi.nlm.nih.gov/pubmed/19302160 19302160 10.1111/j.1460-9568.2009.06688.x

[hbm24715-bib-0029] Feldman, J. I. , Dunham, K. , Cassidy, M. , Wallace, M. T. , Liu, Y. , & Woynaroski, T. G. (2018). Audiovisual multisensory integration in individuals with autism spectrum disorder: A systematic review and meta‐analysis. Neuroscience and Biobehavioral Reviews, 95, 220–234. https://linkinghub.elsevier.com/retrieve/pii/S0149763418303634 30287245 10.1016/j.neubiorev.2018.09.020 PMC6291229

[hbm24715-bib-0030] Foxe, J. J. , Molholm, S. , Del Bene, V. A. , Frey, H. P. , Russo, N. N. , Blanco, D. , … Ross, L. A. (2015). Severe multisensory speech integration deficits in high‐functioning school‐aged children with autism spectrum disorder (ASD) and their resolution during early adolescence. Cerebral Cortex, 25, 298–312. 23985136 10.1093/cercor/bht213 PMC4303800

[hbm24715-bib-0031] Foxe, J. J. , & Schroeder, C. E. (2005). The case for feedforward multisensory convergence during early cortical processing. Neuroreport, 16, 419–423. http://www.ncbi.nlm.nih.gov/pubmed/15770144 15770144 10.1097/00001756-200504040-00001

[hbm24715-bib-0032] Foxton, J. M. , Stewart, M. E. , Barnard, L. , Rodgers, J. , Young, A. H. , O'Brien, G. , & Griffiths, T. D. (2003). Absence of auditory “global interference” in autism. Brain, 126, 2703–2709. https://academic.oup.com/brain/article-lookup/doi/10.1093/brain/awg274 12937074 10.1093/brain/awg274

[hbm24715-bib-0033] Freiherr, J. , Gordon, A. R. , Alden, E. C. , Ponting, A. L. , Hernandez, M. F. , Boesveldt, S. , & Lundström, J. N. (2012). The 40‐item Monell extended Sniffin’ sticks identification test (MONEX‐40). Journal of Neuroscience Methods, 205, 10–16. 22200409 10.1016/j.jneumeth.2011.12.004 PMC3623611

[hbm24715-bib-0034] Friston, K. J. (2005). Models of brain function in neuroimaging. Annual Review of Psychology, 56, 57–87. http://www.annualreviews.org/doi/10.1146/annurev.psych.56.091103.070311 10.1146/annurev.psych.56.091103.070311 15709929

[hbm24715-bib-0035] Friston, K. J. , Harrison, L. , & Penny, W. (2003). Dynamic causal modelling. NeuroImage, 19, 1273–1302. http://www.ncbi.nlm.nih.gov/pubmed/12948688 12948688 10.1016/s1053-8119(03)00202-7

[hbm24715-bib-0036] Frith, U. , & Happé, F. (1994). Autism: Beyond “theory of mind”. Cognition, 50, 115–132. 8039356 10.1016/0010-0277(94)90024-8

[hbm24715-bib-0037] Ghahremani, A. , Rastogi, A. , & Lam, S. (2015). The role of right anterior insula and salience processing in inhibitory control. The Journal of Neuroscience, 35, 3291–3292. http://www.ncbi.nlm.nih.gov/pubmed/25716829 25716829 10.1523/JNEUROSCI.5239-14.2015 PMC6605563

[hbm24715-bib-0038] Gori, M. , Del Viva, M. , Sandini, G. , & Burr, D. C. (2008). Young children do not integrate visual and haptic form information. Current Biology, 18, 694–698. http://www.ncbi.nlm.nih.gov/pubmed/18450446 18450446 10.1016/j.cub.2008.04.036

[hbm24715-bib-0039] Gottfried, J. A. , & Dolan, R. J. (2003). The nose smells what the eye sees: Crossmodal visual facilitation of human olfactory perception. Neuron, 39, 375–386. 12873392 10.1016/s0896-6273(03)00392-1

[hbm24715-bib-0040] Grefkes, C. , & Fink, G. R. (2005). The functional organization of the intraparietal sulcus in humans and monkeys. Journal of Anatomy, 207, 3–17. http://www.ncbi.nlm.nih.gov/pubmed/16011542 16011542 10.1111/j.1469-7580.2005.00426.x PMC1571496

[hbm24715-bib-0041] Grefkes, C. , Weiss, P. H. , Zilles, K. , & Fink, G. R. (2002). Crossmodal processing of object features in human anterior intraparietal cortex: An fMRI study implies equivalencies between humans and monkeys. Neuron, 35, 173–184. https://www.sciencedirect.com/science/article/pii/S0896627302007419?via%3Dihub 12123617 10.1016/s0896-6273(02)00741-9

[hbm24715-bib-0042] Gur, R. C. , Sara, R. , Hagendoorn, M. , Marom, O. , Hughett, P. , Macy, L. , … Gur, R. E. (2002). A method for obtaining 3‐dimensional facial expressions and its standardization for use in neurocognitive studies. Journal of Neuroscience Methods, 115, 137–143. https://www.sciencedirect.com/science/article/pii/S0165027002000067?via%3Dihub#FIG4 11992665 10.1016/s0165-0270(02)00006-7

[hbm24715-bib-0043] Habel, U. , Koch, K. , Pauly, K. , Kellermann, T. , Reske, M. , Backes, V. , … Schneider, F. (2007). The influence of olfactory‐induced negative emotion on verbal working memory: Individual differences in neurobehavioral findings. Brain Research, 1152, 158–170. https://www.sciencedirect.com/science/article/pii/S0006899307006701?via%3Dihub 17448450 10.1016/j.brainres.2007.03.048

[hbm24715-bib-0044] Härtling C., Markowitsch H. J., Neufeld H., Calabrese P., Deisinger K., & Kessler J. (Eds.). (2000). Wechsel Gedächtnis Test, Rev. Fassung (WMS‐R). Bern, Switzerland: Huber.

[hbm24715-bib-0045] Hautzinger, M. , Keller, F. , & Kühner, C. (2006). Beck‐depressions‐inventar revision. Frankfurt, Germany: Pearson.

[hbm24715-bib-0046] Hoheisel, B. , & Kryspin‐Exner, I. (2005). Emotionserkennung in gesichtern und emotionales gesichtergedächtnis. Zeitschrift für Neuropsychologie, 16, 77–87. https://econtent.hogrefe.com/doi/10.1024/1016-264X.16.2.77

[hbm24715-bib-0047] Holmes, N. P. (2009). The principle of inverse effectiveness in multisensory integration: Some statistical considerations. Brain Topography, 21, 168–176. 19404728 10.1007/s10548-009-0097-2

[hbm24715-bib-0048] Hopfinger, J. B. , Buonocore, M. H. , & Mangun, G. R. (2000). The neural mechanisms of top‐down attentional control. Nature Neuroscience, 3, 284–291. http://www.nature.com/articles/nn0300_284 10700262 10.1038/72999

[hbm24715-bib-0049] Kadohisa, M. (2013). Effects of odor on emotion, with implications. Frontiers in Systems Neuroscience, 7, 66 http://www.ncbi.nlm.nih.gov/pubmed/24124415 24124415 10.3389/fnsys.2013.00066 PMC3794443

[hbm24715-bib-0050] Kayser, C. , Logothetis, N. K. , & Panzeri, S. (2010). Visual enhancement of the information representation in auditory cortex. Current Biology, 20, 19–24. https://ac.els-cdn.com/S096098220901940X/1-s2.0-S096098220901940X-main.pdf?_tid=4e1d7348-0e7b-402e-9256-86e8ec99afe4&acdnat=1538039915_1e13c0d7fbb3c72b3dc85d6638d5686a 20036538 10.1016/j.cub.2009.10.068

[hbm24715-bib-0051] Kern, J. K. , Trivedi, M. H. , Grannemann, B. D. , Garver, C. R. , Johnson, D. G. , Andrews, A. A. , … Schroeder, J. L. (2007). Sensory correlations in autism. Autism, 11, 123–134. 17353213 10.1177/1362361307075702

[hbm24715-bib-0052] Kuhl, J. , & Kazén, M. (2009). Persönlichkeits‐Stil und Störungs‐Invenatar (PSSI) (2nd ed.). Göttingen, Germany: Hogrefe.

[hbm24715-bib-0053] Lakatos, P. , Chen, C.‐M. , O'Connell, M. N. , Mills, A. , & Schroeder, C. E. (2007). Neuronal oscillations and multisensory interaction in primary auditory cortex. Neuron, 53, 279–292. http://www.ncbi.nlm.nih.gov/pubmed/17224408 17224408 10.1016/j.neuron.2006.12.011 PMC3717319

[hbm24715-bib-0054] Larsson, M. , Tirado, C. , & Wiens, S. (2017). A meta‐analysis of odor thresholds and odor identification in autism spectrum disorders. Frontiers in Psychology, 8, 1–9. 28553238 10.3389/fpsyg.2017.00679 PMC5425471

[hbm24715-bib-0055] Licon, C. C. , Manesse, C. , Dantec, M. , Fournel, A. , & Bensafi, M. (2018). Pleasantness and trigeminal sensations as salient dimensions in organizing the semantic and physiological spaces of odors. Scientific Reports, 8, 8444 http://www.nature.com/articles/s41598-018-26510-5 29855500 10.1038/s41598-018-26510-5 PMC5981304

[hbm24715-bib-0056] Lundström, J. N. , Gordon, A. R. , Alden, E. C. , & Boesveldt, S. (2011). Methods for building an inexpensive computer‐controlled olfactometer for temporally precise experiments. International Journal of Psychophysiology, 78, 179–189. 10.1016/j.ijpsycho.2010.07.007 PMC2967213 20688109

[hbm24715-bib-0057] Lundström, J. N. , Regenbogen, C. , Ohla, K. , & Seubert, J. (2018). Prefrontal control over occipital responses to crossmodal overlap varies across the congruency spectrum. Cerebral Cortex, 29, 1–11. https://academic.oup.com/cercor/advance-article/doi/10.1093/cercor/bhy168/5060272 10.1093/cercor/bhy168 30060139

[hbm24715-bib-0058] Macaluso, E. (2006). Multisensory processing in sensory‐specific cortical areas. Neuroscience, 12, 327–338. http://journals.sagepub.com/doi/10.1177/1073858406287908 10.1177/1073858406287908 16840709

[hbm24715-bib-0059] Malhotra, P. , Coulthard, E. J. , & Husain, M. (2009). Role of right posterior parietal cortex in maintaining attention to spatial locations over time. Brain, 132, 645–660. http://www.ncbi.nlm.nih.gov/pubmed/19158107 19158107 10.1093/brain/awn350 PMC2664449

[hbm24715-bib-0060] Martzke, J. S. , Kopala, L. C. , & Good, K. P. (1997). Olfactory dysfunction in neuropsychiatric disorders: Review and methodological considerations. Biological Psychiatry, 42, 721–732. http://www.ncbi.nlm.nih.gov/pubmed/9325566 9325566 10.1016/s0006-3223(96)00442-8

[hbm24715-bib-0061] Moessnang, C. , Pauly, K. , Kellermann, T. , Krämer, J. , Finkelmeyer, A. , Hummel, T. , … Habel, U. (2013). The scent of salience–is there olfactory‐trigeminal conditioning in humans? NeuroImage, 77, 93–104. http://www.ncbi.nlm.nih.gov/pubmed/23558094 23558094 10.1016/j.neuroimage.2013.03.049 PMC4132879

[hbm24715-bib-0062] Moscavitch, S.‐D. , Szyper‐Kravitz, M. , & Shoenfeld, Y. (2009). Autoimmune pathology accounts for common manifestations in a wide range of neuro‐psychiatric disorders: The olfactory and immune system interrelationship. Clinical Immunology, 130, 235–243. https://www.sciencedirect.com/science/article/pii/S1521661608008656?via%3Dihub 19097945 10.1016/j.clim.2008.10.010

[hbm24715-bib-0063] Mottron, L. , Dawson, M. , Soulières, I. , Hubert, B. , & Burack, J. (2006). Enhanced perceptual functioning in autism: An update, and eight principles of autistic perception. Journal of Autism and Developmental Disorders, 36, 27–43. http://www.traininautism.com/Mottron/2006Enhancedperceptual%20funct.JADD.pdf 16453071 10.1007/s10803-005-0040-7

[hbm24715-bib-0064] Nagy, A. , Eördegh, G. , Paróczy, Z. , Márkus, Z. , & Benedek, G. (2006). Multisensory integration in the basal ganglia. The European Journal of Neuroscience, 24, 917–924. http://doi.wiley.com/10.1111/j.1460-9568.2006.04942.x 16930419 10.1111/j.1460-9568.2006.04942.x

[hbm24715-bib-0065] Nardini, M. , Bedford, R. , & Mareschal, D. (2010). Fusion of visual cues is not mandatory in children. Proceedings of the National Academy of Sciences of the United States of America, 107, 17041–17046. http://www.ncbi.nlm.nih.gov/pubmed/20837526 20837526 10.1073/pnas.1001699107 PMC2947870

[hbm24715-bib-0066] Naudin, M. , & Atanasova, B. (2014). Olfactory markers of depression and Alzheimer's disease. Neuroscience and Biobehavioral Reviews, 45, 262–270. 25003804 10.1016/j.neubiorev.2014.06.016

[hbm24715-bib-0067] Nourski, K. V. (2017). Auditory processing in the human cortex: An intracranial electrophysiology perspective. Laryngoscope Investigative Otolaryngology, 2, 147–156. http://doi.wiley.com/10.1002/lio2.73 28894834 10.1002/lio2.73 PMC5562943

[hbm24715-bib-0068] Orr, J. M. , & Weissman, D. H. (2009). Anterior cingulate cortex makes 2 contributions to minimizing distraction. Cerebral Cortex, 19, 703–711. http://www.ncbi.nlm.nih.gov/pubmed/18653665 18653665 10.1093/cercor/bhn119 PMC2637305

[hbm24715-bib-0069] Pellicano, E. (2010). The development of core cognitive skills in autism: A 3‐year prospective study. Child Development, 81, 1400–1416. http://www.ncbi.nlm.nih.gov/pubmed/20840230 20840230 10.1111/j.1467-8624.2010.01481.x

[hbm24715-bib-1005] Pessoa, L. , Japee, S. , Sturman, D. , & Ungerleider, L. G. (2006). Target visibility and visual awareness mnodulate amygdala responses to fearful faces. Cereb Cortex, 16, 366–375. 15930371 10.1093/cercor/bhi115

[hbm24715-bib-0070] Posner, M. I. , Nissen, M. J. , & Klein, R. M. (1976). Visual dominance: An information‐processing account of its origins and significance. Psychological Review, 83, 157–171. http://doi.apa.org/getdoi.cfm?doi=10.1037/0033-295X.83.2.157 769017

[hbm24715-bib-0071] Regenbogen, C. , Habel, U. , & Kellermann, T. (2013). Connecting multimodality in human communication. Frontiers in Human Neuroscience, 7, 754 http://www.ncbi.nlm.nih.gov/pubmed/24265613 24265613 10.3389/fnhum.2013.00754 PMC3820976

[hbm24715-bib-0072] Regenbogen, C. , Seubert, J. , Johansson, E. , Finkelmeyer, A. , Andersson, P. , & Lundström, J. N. (2017). The intraparietal sulcus governs multisensory integration of audiovisual information based on task difficulty. Human Brain Mapping, 39, 1313–1326. http://doi.wiley.com/10.1002/hbm.23918 29235185 10.1002/hbm.23918 PMC6866436

[hbm24715-bib-0073] Reitan, R. M. (1992). Trail making test. Tucson, AZ: Reitan Neuropsychology Laboratory.

[hbm24715-bib-0074] Renier, L. A. , Anurova, I. , De Volder, A. G. , Carlson, S. , VanMeter, J. , & Rauschecker, J. P. (2009). Multisensory integration of sounds and vibrotactile stimuli in processing streams for “what” and “where”. The Journal of Neuroscience, 29, 10950–10960. 19726653 10.1523/JNEUROSCI.0910-09.2009 PMC3343457

[hbm24715-bib-0075] Ripp, I. , zur Nieden, A. N. , Blankenagel, S. , Franzmeier, N. , Lundström, J. N. , & Freiherr, J. (2018). Multisensory integration processing during olfactory‐visual stimulation—An fMRI graph theoretical network analysis. Human Brain Mapping, 39, 1–15. 10.1002/hbm.24206 PMC6866557 29736907

[hbm24715-bib-0076] Rolls, E. T. , Grabenhorst, F. , & Parris, B. A. (2010). Neural systems underlying decisions about affective odors. Journal of Cognitive Neuroscience, 22, 1069–1082. 19320548 10.1162/jocn.2009.21231

[hbm24715-bib-0077] Royet, J. P. , Plailly, J. , Delon‐Martin, C. , Kareken, D. A. , & Segebarth, C. (2003). fMRI of emotional responses to odors: Influence of hedonic valence and judgment, handedness, and gender. NeuroImage, 20, 713–728. 14568446 10.1016/S1053-8119(03)00388-4

[hbm24715-bib-0078] Rozenkrantz, L. , Zachor, D. , Heller, I. , Plotkin, A. , Weissbrod, A. , Snitz, K. , … Sobel, N. (2015). A mechanistic link between olfaction and autism spectrum disorder. Current Biology, 25, 1904–1910. 10.1016/j.cub.2015.05.048 26144969 PMC4518448

[hbm24715-bib-0079] Rühl, D. , Bölte, S. , Feineis‐Matthews, S. , & Poustka, F. (2004). ADOS Diagnostische Beobachtungsskala für Autistische Störungen. Bern, Switzerland: Huber. 10.1024/1422-4917.32.1.45 14992047

[hbm24715-bib-0080] Salomon, R. , Ronchi, R. , Dönz, J. , Bello‐Ruiz, J. , Herbelin, B. , Martet, R. , … Blanke, O. (2016). The insula mediates access to awareness of visual stimuli presented synchronously to the heartbeat. The Journal of Neuroscience, 36, 5115–5127. http://www.ncbi.nlm.nih.gov/pubmed/27147663 27147663 10.1523/JNEUROSCI.4262-15.2016 PMC6601849

[hbm24715-bib-0081] Schmidt, K. H. , & Metzler, P. (1992). Wortschatztest. Beltz Test: Weinheim, Germany.

[hbm24715-bib-0082] Schneider, T. R. , Engel, A. K. , & Debener, S. (2008). Multisensory identification of natural objects in a two‐way crossmodal priming paradigm. Experimental Psychology, 55, 121–132. 18444522 10.1027/1618-3169.55.2.121

[hbm24715-bib-0083] Schoenbaum, G. , Chiba, A. A. , & Gallagher, M. (1999). Neural encoding in orbitofrontal cortex and basolateral amygdala during olfactory discrimination learning. The Journal of Neuroscience, 19, 1876–1884. http://www.ncbi.nlm.nih.gov/pubmed/10024371 10024371 10.1523/JNEUROSCI.19-05-01876.1999 PMC6782178

[hbm24715-bib-0084] Schulte‐Rüther, M. , Greimel, E. , Piefke, M. , Kamp‐Becker, I. , Remschmidt, H. , Fink, G. R. , … Konrad, K. (2014). Age‐dependent changes in the neural substrates of empathy in autism spectrum disorder. Social Cognitive and Affective Neuroscience, 9, 1118–1126. 23784073 10.1093/scan/nst088 PMC4127013

[hbm24715-bib-0085] Selemon, L. D. (2013). A role for synaptic plasticity in the adolescent development of executive function. Translational Psychiatry, 3, e238–e239. 10.1038/tp.2013.7 23462989 PMC3625918

[hbm24715-bib-0086] Sereno, M. I. , & Huang, R.‐S. (2014). Multisensory maps in parietal cortex. Current Opinion in Neurobiology, 24, 39–46. https://www.sciencedirect.com/science/article/pii/S0959438813001736 24492077 10.1016/j.conb.2013.08.014 PMC3969294

[hbm24715-bib-0087] Seubert, J. , Freiherr, J. , Djordjevic, J. , & Lundström, J. N. (2013). Statistical localization of human olfactory cortex. NeuroImage, 66, 333–342. http://www.ncbi.nlm.nih.gov/pubmed/23103688 23103688 10.1016/j.neuroimage.2012.10.030

[hbm24715-bib-0088] Seubert, J. , Kellermann, T. , Loughead, J. , Boers, F. , Brensinger, C. , Schneider, F. , & Habel, U. (2010). Processing of disgusted faces is facilitated by odor primes: A functional MRI study. NeuroImage, 53, 746–756. 10.1016/j.neuroimage.2010.07.012 20627130

[hbm24715-bib-0089] Sijben, R. , Hoffmann‐Hensel, S. M. , Rodrigues‐Raecke, R. , Haarmeier, T. , & Freiherr, J. (2018). Semantic congruence alters functional connectivity during olfactory‐visual perception. Chemical Senses, 43, 599–610. 30010874 10.1093/chemse/bjy048

[hbm24715-bib-0090] Stein, B. E. , & Stanford, T. R. (2008). Multisensory integration: Current issues from the perspective of the single neuron. Nature Reviews. Neuroscience, 9, 255–266. 18354398 10.1038/nrn2331

[hbm24715-bib-0091] Stein, B. E. , Stanford, T. R. , & Rowland, B. A. (2014). Development of multisensory integration from the perspective of the individual neuron. Nature Reviews. Neuroscience, 15, 520–535. 25158358 10.1038/nrn3742 PMC4215474

[hbm24715-bib-0092] Stephan, K. E. , Penny, W. D. , Daunizeau, J. , Moran, R. J. , & Friston, K. J. (2009). Bayesian model selection for group studies. NeuroImage, 46, 1004–1017. http://www.ncbi.nlm.nih.gov/pubmed/19306932 19306932 10.1016/j.neuroimage.2009.03.025 PMC2703732

[hbm24715-bib-0093] Stephan, K. E. , Penny, W. D. , Moran, R. J. , den Ouden, H. E. M. , Daunizeau, J. , & Friston, K. J. (2010). Ten simple rules for dynamic causal modeling. NeuroImage, 49, 3099–3109. http://www.ncbi.nlm.nih.gov/pubmed/19914382 19914382 10.1016/j.neuroimage.2009.11.015 PMC2825373

[hbm24715-bib-0094] Stevenson, R. A. , Geoghegan, M. L. , & James, T. W. (2007). Superadditive BOLD activation in superior temporal sulcus with threshold non‐speech objects. Experimental Brain Research, 179, 85–95. http://www.ncbi.nlm.nih.gov/pubmed/17109108 17109108 10.1007/s00221-006-0770-6

[hbm24715-bib-0095] Stevenson, R. A. , Ghose, D. , Fister, J. K. , Sarko, D. K. , Altieri, N. A. , Nidiffer, A. R. , … Wallace, M. T. (2014). Identifying and quantifying multisensory integration: A tutorial review. Brain Topography, 27, 707–730. http://link.springer.com/10.1007/s10548-014-0365-7 24722880 10.1007/s10548-014-0365-7

[hbm24715-bib-0096] Stevenson, R. A. , Siemann, J. K. , Schneider, B. C. , Eberly, H. E. , Woynaroski, T. G. , Camarata, S. M. , & Wallace, M. T. (2014). Multisensory temporal integration in autism spectrum disorders. The Journal of Neuroscience, 34, 691–697. http://www.jneurosci.org/cgi/doi/10.1523/JNEUROSCI.3615-13.2014 24431427 10.1523/JNEUROSCI.3615-13.2014 PMC3891950

[hbm24715-bib-0097] Suzuki, Y. (2003). Impaired olfactory identification in Asperger's syndrome. Journal of Neuropsychiatry, 15, 105–107. http://neuro.psychiatryonline.org/article.aspx?articleID=101770 10.1176/jnp.15.1.105 12556580

[hbm24715-bib-0098] Taylor, N. , Isaac, C. , & Milne, E. (2010). A comparison of the development of audiovisual integration in children with autism spectrum disorders and typically developing children. Journal of Autism and Developmental Disorders, 40, 1403–1411. http://link.springer.com/10.1007/s10803-010-1000-4 20354776 10.1007/s10803-010-1000-4

[hbm24715-bib-0099] Thye, M. D. , Bednarz, H. M. , Herringshaw, A. J. , Sartin, E. B. , & Kana, R. K. (2018). The impact of atypical sensory processing on social impairments in autism spectrum disorder. Developmental Cognitive Neuroscience, 29, 151–167. 10.1016/j.dcn.2017.04.010 28545994 PMC6987885

[hbm24715-bib-0100] Tonacci, A. , Billeci, L. , Tartarisco, G. , Ruta, L. , Muratori, F. , Pioggia, G. , & Gangemi, S. (2015). Olfaction in autism spectrum disorders: A systematic review. Child Neuropsychology, 7049, 1–25. 10.1080/09297049.2015.1081678 26340690

[hbm24715-bib-0101] Turetsky, B. I. , Hahn, C.‐G. , Borgmann‐Winter, K. , & Moberg, P. J. (2009). Scents and nonsense: Olfactory dysfunction in schizophrenia. Schizophrenia Bulletin, 35, 1117–1131. http://www.ncbi.nlm.nih.gov/pubmed/19793796 19793796 10.1093/schbul/sbp111 PMC2762633

[hbm24715-bib-0102] Weisberg, J. , Hubbard, A. L. , & Emmorey, K. (2017). Multimodal integration of spontaneously produced representational co‐speech gestures: An fMRI study. Language, Cognition and Neuroscience, 32, 158–174. http://www.ncbi.nlm.nih.gov/pubmed/29130054 10.1080/23273798.2016.1245426 PMC5675577 29130054

[hbm24715-bib-0103] Weiss, E. M. , Walter, C. , Fink, A. , Schulter, G. , Mittenecker, E. , & Papousek, I. (2018). Age‐moderating effect in prepotent response inhibition in boys with Asperger syndrome: A 2.5 years longitudinal study. European Archives of Psychiatry and Clinical Neuroscience, 269, 1–4. 10.1007/s00406-018-0915-1 PMC6469602 29942979

[hbm24715-bib-0104] Wicker, B. , Monfardini, E. , & Royet, J.‐P. (2016). Olfactory processing in adults with autism spectrum disorders. Molecular Autism, 7, 4 http://www.molecularautism.com/content/7/1/4 26788281 10.1186/s13229-016-0070-3 PMC4717566

[hbm24715-bib-0105] Wittchen, H.‐U. , Zaudig, M. , & Frydrich, T. (1997). Strukuriertes Klinisches Interview für DSM‐IV. Göttingen, Germany: Hogrefe.

[hbm24715-bib-0106] Yuan, T.‐F. , & Slotnick, B. M. (2014). Roles of olfactory system dysfunction in depression. Progress in Neuro‐Psychopharmacology & Biological Psychiatry, 54, 26–30. 24879990 10.1016/j.pnpbp.2014.05.013

[hbm24715-bib-0107] Zald, D. H. , & Pardo, J. V. (1997). Emotion, olfaction, and the human amygdala: Amygdala activation during aversive olfactory stimulation. Proceedings of the National Academy of Sciences of the United States of America, 94, 4119–4124. 9108115 10.1073/pnas.94.8.4119 PMC20578

[hbm24715-bib-0108] Zelano, C. , Bensafi, M. , Porter, J. , Mainland, J. , Johnson, B. , Bremner, E. , … Sobel, N. (2005). Attentional modulation in human primary olfactory cortex. Nature Neuroscience, 8, 114–120. http://www.ncbi.nlm.nih.gov/pubmed/15608635 15608635 10.1038/nn1368

