# Supplementary material for: Audio–visual and olfactory–visual integration in healthy participants and subjects with autism spectrum disorder
Source: Hum Brain Mapp. 2019 Jul 13;40(15):4470–86. doi: 10.1002/hbm.24715 (PMC6865810; doi:10.1002/hbm.24715)
Supplement: Supplementary file 1 — Figure S1 Mean pleasantness ratings and standard errors (error bar) across both groups and both experiment. Figure S2. Family‐level inference with linear and nonlinear models, across both groups and both experiments. Table S1 Means and standard deviation of the pleasantness rating in unimodal and bimodal combination for both tasks (olfactory–visual and auditory–visual stimulation). Table S2 Free energy of all models across all subjects and both experiments, and model in Occam's window for all subjects. [file HBM-40-4470-s001.docx]

**Supplementary Material**

**Behavioral results**

During the olfactory-visual stimulation, we found no significant effect of the group, F(1, 33) = 1.23, p = .275, eta^2^ = .036. We found a significant effect of the factor pleasantness, F(1, 33) = 55.375, p < .001, eta^2^ = .627, reflected higher pleasantness rating for pleasant compared to unpleasant odors (p < .001). An interaction of pleasantness x congruency, F(2, 66) = 13.183, p < .001, eta^2^ = .285, emerged (Figure S1). In the pleasant condition congruent odor-picture (OPVP) combination were rated as more pleasant than incongruent (OPVU) combination (p = .005), and unimodal (OP) stimulation (p = .002) (Table S1). In the unpleasant condition incongruent odor-picture (OUVP) combination were rated as more pleasant than congruent (OUVU) and unimodal (OU) stimulation (p < .001, p = .008) (Table S1).

During the auditory-visual stimulation, we found no significant effect of the group, F(1, 33) = 0.15, p = .701, eta^2^ = .005. We found a significant effect of the factor pleasantness, F(1, 33) = 52.68, p < .001, eta^2^ = .615, with higher pleasantness rating for pleasant audios compared to unpleasant audios (p < .001). An interaction of pleasantness x congruency, F(1.616, 53.316) = 5.337, p = .012 eta^2^ = .139, emerged. In the pleasant condition congruent odor-picture (APVP) combination were rated as more pleasant than incongruent (APVU) combination (p = .002), and unimodal (OP) stimulation (p = .002) (Table S1).


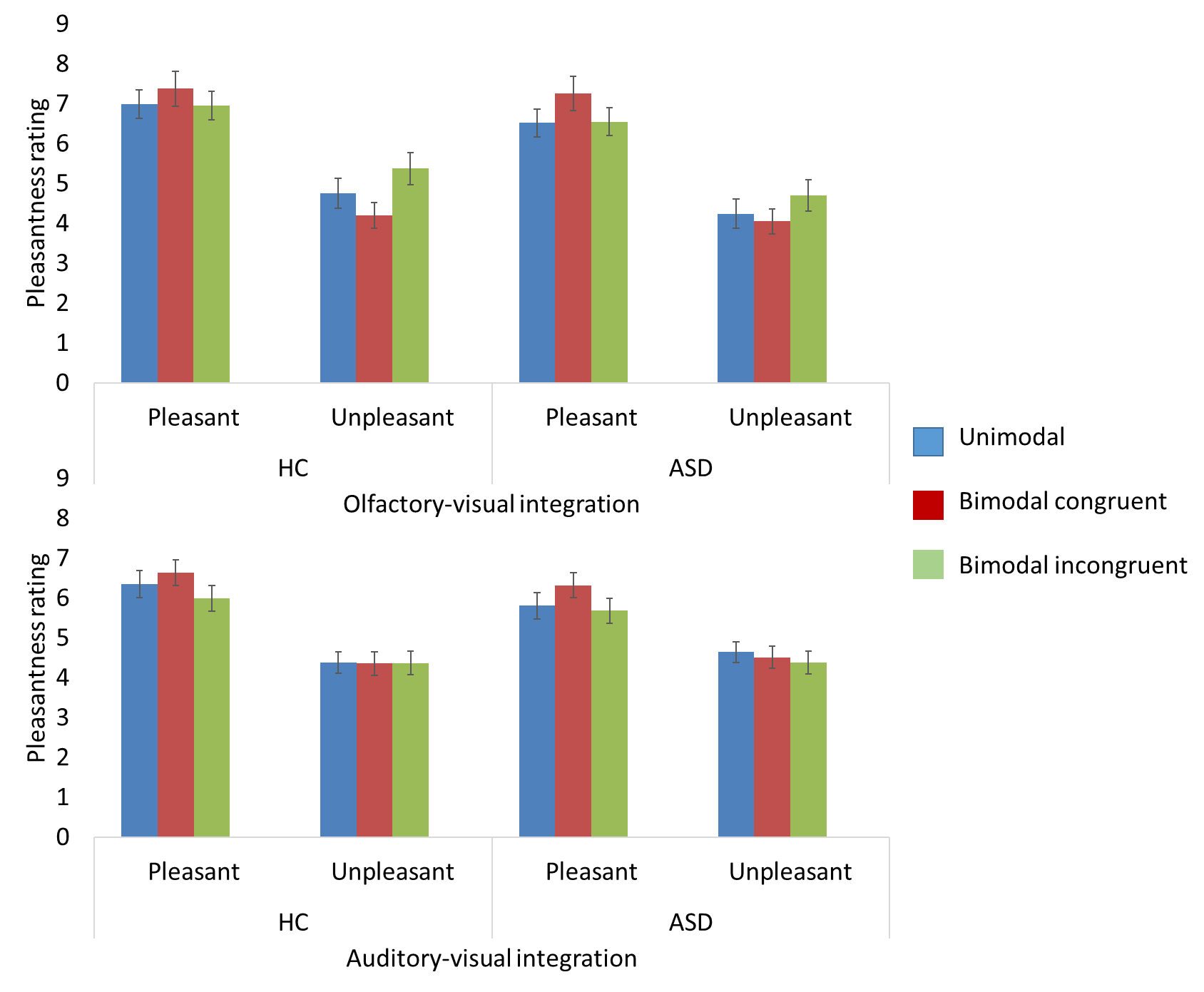


Figure S1. Mean pleasantness ratings and standard errors (error bar) across both groups and both experiment.

Table S1

Means and standard deviation of the pleasantness rating in unimodal and bimodal combination for both tasks (olfactory-visual and auditory-visual stimulation).

|  |  | ASD | HC |
| --- | --- | --- | --- |
|  |  | Mean (SD) | Mean (SD) |
| Pleasant Odor | Unimodal | 6.51 (1.39) | 6.98 (1.51) |
|  | Bimodal congruent | 7.25 (1.75) | 7.37 (1.83) |
|  | Bimodal incongruent | 6.54 (1.41) | 6.95 (1.49) |
| Unpleasant Odor | Unimodal | 4.24 (1.30) | 4.75 (1.77) |
|  | Bimodal congruent | 4.05 (1.12) | 4.19 (1.49) |
|  | Bimodal incongruent | 4.69 (1.41) | 5.37 (1.89) |
| Pleasant Audio | Unimodal | 5.80 (1.34) | 6.35 (1.44) |
|  | Bimodal congruent | 6.31 (1.29) | 6.63 (1.32) |
|  | Bimodal incongruent | 5.68 (1.11) | 5.98 (1.50) |
| Unpleasant Audio | Unimodal | 4.63 (0.89) | 4.37 (1.28) |
|  | Bimodal congruent | 4.50 (1.07) | 4.35 (1.31) |
|  | Bimodal incongruent | 4.37 (1.03) | 4.36 (1.34) |

Table S2

Free energy of all models across all subjects and both experiments, and model in Occam’s window for all subjects.

|  |  | Free energy | | | | | Models in Occam's window |
| --- | --- | --- | --- | --- | --- | --- | --- |
|  |  | Model 1 | Model 2 | Model 3 | Model 4 | Model 5 |  |
| **Auditory-visual integration** | |  |  |  |  |  |  |
|  | **ASD** |  |  |  |  |  |  |
|  | Subject 1 | -5.22^3^ | -5.37^3^ | -5.37^3^ | -5.17^3^ | -5.35^3^ | 4 |
|  | Subject 2 | -2.93^3^ | -2.93^3^ | -2.93^3^ | -2.93^3^ | -2.93^3^ | 4 |
|  | Subject 3 | -3.70^3^ | -3.72^3^ | -3.72^3^ | -3.68^3^ | -3.70^3^ | 4 |
|  | Subject 4 | -4.85^3^ | -4.84^3^ | -4.84^3^ | -4.82^3^ | -4.81^3^ | 5 |
|  | Subject 5 | -5.57^3^ | -5.57^3^ | -5.57^3^ | -5.32^3^ | -5.31^3^ | 5 |
|  | Subject 6 | -4.71^3^ | -4.71^3^ | -4.71^3^ | -4.68^3^ | -4.68^3^ | 4, 5 |
|  | Subject 7 | -5.56^3^ | -5.57^3^ | -5.57^3^ | -5.51^3^ | -5.50^3^ | 5 |
|  | Subject 8 | -5.22^3^ | -5.23^3^ | -5.23^3^ | -5.17^3^ | -5.18^3^ | 4 |
|  | Subject 9 | -2.35^3^ | -2.35^3^ | -2.35^3^ | -2.34^3^ | -2.33^3^ | 4, 5 |
|  | Subject 10 | -5.02^3^ | -5.03^3^ | -5.03^3^ | -5.01^3^ | -5.01^3^ | 4 |
|  | Subject 11 | -4.84^3^ | -4.84^3^ | -4.84^3^ | -4.81^3^ | -4.81^3^ | 5 |
|  | Subject 12 | -4.73^3^ | -4.75^3^ | -4.75^3^ | -4.65^3^ | -4.70^3^ | 4 |
|  | Subject 13 | -4.25^3^ | -4.25^3^ | -4.25^3^ | -4.23^3^ | -4.23^3^ | 4 |
|  | Subject 14 | -3.49^3^ | -3.49^3^ | -3.49^3^ | -3.49^3^ | -3.49^3^ | 4, 5 |
|  | Subject 15 | -2.96^3^ | -2.95^3^ | -2.95^3^ | -2.94^3^ | -2.93^3^ | 4, 5 |
|  | Subject 16 | -4.95^3^ | -4.95^3^ | -4.95^3^ | -4.91^3^ | -4.92^3^ | 4 |
|  | Subject 17 | -4.90^3^ | -4.92^3^ | -4.92^3^ | -4.92^3^ | -4.79^3^ | 5 |
|  | Subject 18 | -1.42^3^ | -1.42^3^ | -1.42^3^ | -1.39^3^ | -1.39^3^ | 4 |
|  | **HC** |  |  |  |  |  |  |
|  | Subject 1 | -4.52^3^ | -4.53^3^ | -4.55^3^ | -4.50^3^ | -4.53^3^ | 4 |
|  | Subject 2 | -4.46^3^ | -4.47^3^ | -4.46^3^ | -4.40^3^ | -4.43^3^ | 4 |
|  | Subject 3 | -4.93^3^ | -4.92^3^ | -4.92^3^ | -4.85^3^ | -4.84^3^ | 5 |
|  | Subject 4 | -4.15^3^ | -4.18^3^ | -4.18^3^ | -4.12^3^ | -4.13^3^ | 4 |
|  | Subject 5 | -1.38^3^ | -1.39^3^ | -1.39^3^ | -1.38^3^ | -1.38^3^ | 4, 5 |
|  | Subject 6 | -2.25^3^ | -2.25^3^ | -2.25^3^ | -2.23^3^ | -2.23^3^ | 4, 5 |
|  | Subject 7 | -5.15^3^ | -5.19^3^ | -5.19^3^ | -5.09^3^ | -5.14^3^ | 4 |
|  | Subject 8 | -3.48^3^ | -3.51^3^ | -3.51^3^ | -3.44^3^ | -3.50^3^ | 4 |
|  | Subject 9 | -4.86^3^ | -4.86^3^ | -4.86^3^ | -4.84^3^ | -4.84^3^ | 4 |
|  | Subject 10 | -4.00^3^ | -4.00^3^ | -4.00^3^ | -3.97^3^ | -3.97^3^ | 4 |
|  | Subject 11 | -4.56^3^ | -4.57^3^ | -4.57^3^ | -4.52^3^ | -4.53^3^ | 4 |
|  | Subject 12 | -4.83^3^ | -4.86^3^ | -4.86^3^ | -4.77^3^ | -4.82^3^ | 4 |
|  | Subject 13 | -4.90^3^ | -4.92^3^ | -4.92^3^ | -4.92^3^ | -4.79^3^ | 5 |
|  | Subject 14 | -4.64^3^ | -4.64^3^ | -4.64^3^ | -4.56^3^ | -4.59^3^ | 4 |
|  | Subject 15 | -4.73^3^ | -4.73^3^ | -4.73^3^ | -4.72^3^ | -4.71^3^ | 4, 5 |
|  | Subject 16 | -4.92^3^ | -4.91^3^ | -4.91^3^ | -4.89^3^ | -4.89^3^ | 4 |
|  | Subject 17 | -2.02^3^ | -2.02^3^ | -2.02^3^ | -2.01^3^ | -2.01^3^ | 4, 5 |
|  |  |  |  |  |  |  |  |
| **Olfactory-visual integration** | |  |  |  |  |  |  |
|  | **ASD** |  |  |  |  |  |  |
|  | Subject 1 | -4.09^3^ | -4.13^3^ | -4.13^3^ | -4.09^3^ | -4.13^3^ | 4 |
|  | Subject 2 | -4.13^3^ | -4.13^3^ | -4.13^3^ | -4.13^3^ | -4.13^3^ | 1, 2, 3, 4, 5 |
|  | Subject 3 | -2.68^3^ | -2.70^3^ | -2.70^3^ | -2.67^3^ | -2.70^3^ | 4 |
|  | Subject 4 | -4.67^3^ | -4.68^3^ | -4.68^3^ | -4.66^3^ | -4.67^3^ | 4 |
|  | Subject 5 | -4.95^3^ | -4.99^3^ | -4.99^3^ | -4.90^3^ | -4.96^3^ | 4 |
|  | Subject 6 | -2.01^3^ | -2.02^3^ | -2.02^3^ | -2.01^3^ | -2.02^3^ | 4 |
|  | Subject 7 | -5.32^3^ | -5.33^3^ | -5.33^3^ | -5.30^3^ | -5.32^3^ | 4 |
|  | Subject 8 | -6.07^3^ | -6.08^3^ | -6.08^3^ | -6.06^3^ | -6.08^3^ | 4 |
|  | Subject 9 | -4.58^3^ | -4.58^3^ | -4.58^3^ | -4.56^3^ | -4.57^3^ | 4 |
|  | Subject 10 | -4.44^3^ | -4.44^3^ | -4.44^3^ | -4.41^3^ | -4.41^3^ | 4, 5 |
|  | Subject 11 | -4.75^3^ | -4.75^3^ | -4.75^3^ | -4.72^3^ | -4.72^3^ | 4, 5 |
|  | Subject 12 | -4.18^3^ | -4.17^3^ | -4.17^3^ | -4.13^3^ | -4.12^3^ | 5 |
|  | Subject 13 | -6.14^2^ | -6.11^2^ | -6.11^2^ | -6.06^2^ | -6.03^2^ | 4, 5 |
|  | Subject 14 | -4.85^3^ | -4.85^3^ | -4.85^3^ | -4.81^3^ | -4.81^3^ | 4, 5 |
|  | Subject 15 | -4.67^3^ | -4.67^3^ | -4.67^3^ | -4.67^3^ | -4.64^3^ | 5 |
|  | Subject 16 | -4.60^3^ | -4.67^3^ | -4.68^3^ | -4.59^3^ | -4.67^3^ | 4 |
|  | Subject 17 | -5.32^3^ | -5.32^3^ | -5.32^3^ | -5.28^3^ | -5.31^3^ | 4 |
|  | Subject 18 | -4.45^3^ | -4.44^3^ | -4.44^3^ | -4.44^3^ | -4.44^3^ | 4, 5 |
|  | **HC** |  |  |  |  |  |  |
|  | Subject 1 | -5.75^3^ | -5.75^3^ | -5.75^3^ | -5.72^3^ | -5.74^3^ | 4 |
|  | Subject 2 | -5.48^3^ | -5.48^3^ | -5.48^3^ | -5.46^3^ | -5.46^3^ | 4 |
|  | Subject 3 | -4.54^3^ | -4.54^3^ | -4.54^3^ | -4.54^3^ | -4.54^3^ | 4, 5 |
|  | Subject 4 | -4.33^3^ | -4.32^3^ | -4.32^3^ | -4.32^3^ | -4.32^3^ | 4, 5 |
|  | Subject 5 | -2.42^3^ | -2.42^3^ | -2.42^3^ | -2.42^3^ | -2.42^3^ | 5 |
|  | Subject 6 | -4.59^3^ | -4.60^3^ | -4.60^3^ | -4.60^3^ | -4.60^3^ | 1, 4 |
|  | Subject 7 | -5.28^3^ | -5.28^3^ | -5.28^3^ | -5.26^3^ | -5.26^3^ | 5 |
|  | Subject 8 | -4.80^3^ | -4.80^3^ | -4.80^3^ | -4.79^3^ | -4.79^3^ | 4, 5 |
|  | Subject 9 | -5.50^3^ | -5.50^3^ | -5.50^3^ | -5.47^3^ | -5.46^3^ | 5 |
|  | Subject 10 | -5.58^3^ | -5.61^3^ | -5.61^3^ | -5.56^3^ | -5.61^3^ | 4 |
|  | Subject 11 | -5.03^3^ | -5.03^3^ | -5.03^3^ | -5.02^3^ | -5.02^3^ | 4 |
|  | Subject 12 | -3.90^3^ | -3.90^3^ | -3.90^3^ | -3.89^3^ | -3.89^3^ | 4, 5 |
|  | Subject 13 | -5.32^3^ | -5.32^3^ | -5.32^3^ | -5.28^3^ | -5.31^3^ | 4 |
|  | Subject 14 | -4.45^3^ | -4.46^3^ | -4.46^3^ | -4.44^3^ | -4.44^3^ | 4 |
|  | Subject 15 | -3.76^3^ | -3.78^3^ | -3.78^3^ | -3.74^3^ | -3.77^3^ | 4 |
|  | Subject 16 | -5.05^3^ | -5.07^3^ | -5.07^3^ | -5.03^3^ | -5.07^3^ | 4 |
|  | Subject 17 | -1.65^3^ | -1.65^3^ | -1.65^3^ | -1.64^3^ | -1.64^3^ | 4, 5 |

Note. Free energy is rounded to two decimal points.


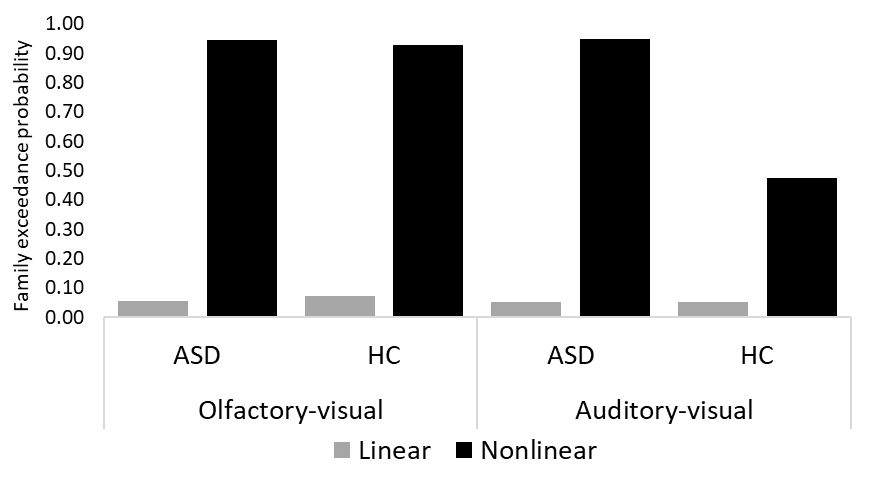


Figure S2. Family-level inference with linear and nonlinear models, across both groups and both experiments.
